# Machine learning-based detection of cognitive decline using SSWTRT: classification performance and decision analysis

**DOI:** 10.3389/frai.2025.1689182

**Published:** 2025-10-29

**Authors:** Yuji Nozaki, Chihiro Kamohara, Ryota Abe, Taiki Ieda, Madoka Nakajima, Maki Sakamoto

**Affiliations:** 1 Department of Informatics, Graduate School of Informatics and Engineering, The University of Electro-Communications, Chofu, Japan; 2 Research Institute for Diseases of Old Age, Juntendo University School of Medicine, Tokyo, Japan; 3 Department of Neurosurgery, Juntendo University School of Medicine, Tokyo, Japan

**Keywords:** sound symbolic words, texture recognition, dementia, neuropsychological tests, machine learning, SHAP

## Abstract

**Introduction:**

Early detection of cognitive decline is essential for preventing dementia progression, yet conventional screening tools such as the Mini-Mental State Examination (MMSE) require trained examiners and substantial time. Building on evidence that dementia is associated with tactile and visual perceptual deficits, this study examined whether the *Sound Symbolic Word Texture Recognition Test* (SSWTRT)—a rapid, self-administered task using Japanese sound-symbolic words (SSWs)—could identify individuals with suspected cognitive decline through machine learning analysis.

**Methods:**

A total of 233 participants diagnosed with idiopathic normal pressure hydrocephalus (mean age = 77.1 ± 7.3 years) completed the SSWTRT, which presents 12 close-up images of material surfaces and requires selecting one of eight SSWs to describe perceived texture. Each response was scored by its concordance with normative data from healthy young adults. Using these 12 item scores, together with participants’ age and education, several machine learning classifiers were trained to predict MMSE-based groups (≤27 vs. ≥28). Model performance was evaluated via five-fold cross-validation, and interpretability was examined using SHapley Additive exPlanations (SHAP).

**Results:**

Among the tested models—K-Nearest Neighbors, Random Forest, and Support Vector Machine (SVM)—the balanced SVM achieved the highest performance (accuracy = 0.71, precision = 0.72, recall = 0.72, F1 = 0.72, AUC = 0.72). SHAP analysis revealed that responses to specific images, especially those depicting soft or coarse textures, strongly influenced classification outcomes. Some image items showed effects opposite to the intended scoring direction, indicating possible interference from age-related sensory decline rather than cognitive factors.

**Discussion:**

These findings demonstrate that machine learning applied to SSWTRT responses can moderately classify individuals with potential cognitive decline using a non-invasive, resource-efficient approach. The model’s interpretability analysis highlighted key image features and response tendencies associated with cognitive status, providing guidance for test refinement. Although the current cohort consisted solely of iNPH patients, limiting generalizability, the proposed framework offers a promising foundation for scalable, language-specific cognitive screening tools.

## 1 Introduction

In response to the increasing number of elderly individuals with dementia due to population aging, dementia measures have recently been prioritized as one of the most critical issues in social security policies in advanced countries. Early detection of cognitive decline, including mild cognitive impairment (MCI), which lies between normal cognitive aging and dementia, and the implementation of appropriate interventions, may prevent the onset of dementia (Livingston et al., 2024; Ngandu et al., 2015; Cooper et al., 2024). Therefore, the early detection of cognitive decline is crucial in dementia countermeasures.

The Mini-Mental State Examination (MMSE) (Folstein et al., 1975) is one of the most widely used screening tests for dementia. However, its implementation requires assistance from trained professionals, posing challenges to widespread and cost-effective deployment (Sakamoto, 2016). Additionally, patients may be reluctant to undergo cognitive function tests due to concerns about potential cognitive impairment or fear of poor performance. Therefore, the development of an easy-to-administer cognitive screening test that allows patients to take it comfortably and can detect the early stages of dementia would be beneficial.

To develop a test for detecting cognitive decline in people with dementia, we focused on texture recognition abilities, which are essential in everyday life. Previous studies have shown that people with Lewy body dementia and Alzheimer’s disease differ from those without in their ability to recognize the texture of images presented to them, particularly in their difficulty distinguishing between wet and shiny objects in photographs (Oishi et al., 2018). In addition, it was reported that their perception of the freshness of vegetables through texture perception was significantly reduced (Oishi et al., 2020). Moreover, several previous studies have reported that dementia patients experience a decline in texture recognition ability (Battelli et al., 1997; Cavina-Pratesi et al., 2010; Bassi et al., 1993).

However, only a few studies have focused specifically on texture recognition ability. Against this background, we recently reported a method to test how subjects recognize the surface texture of common objects, either through verbal expressions or from photographs of the objects (Kamohara et al., 2024).

When communicating the textures to others, sound symbolic words (SSWs) are often used, especially among Japanese people. In this context, synesthetic associations between sounds and sensory experiences (sound symbolism) have been proven for several decades (Jespersen, 1921; Newman, 1933; Taylor, 1963; Werner and Wapner, 1952; Brown et al., 1955; Hinton et al., 2006; Nuckolls, 1999; Wertheimer, 1958; Sapir, 1929).

Regarding the cross-modal correspondence between sounds and visual shapes shown by studies Ramachandran and Hubbard (2001), Köhler (1929), and Maurer et al. (2006), words such as “marma” and “bouba” tend to be associated with round shapes, while words such as “takete” and “kiki” tend to be associated with angular shapes.

In addition, several recent studies have shown the relationship between the iconic sounds of sounds and the sense of touch (Wong et al., 2022; Sakamoto and Watanabe, 2018).

SSWs, or onomatopoeias as they are commonly called, are the verbalization of auditory information from the environment. A previous study by Hashimoto et al. (2014) showed that SSWs are more frequently used by aphasic patients than healthy subjects and are less likely to be affected by aphasia symptoms, and some recent studies have also shown a link between the symbolic sound of sounds and the tactile sensation (Dingemanse and Majid, 2012).

Motivated by these previous studies, we developed a screening test named the Sound Symbolic Words Texture Recognition Test (SSWTRT) (Kamohara et al., 2024) aimed at the early detection of mild dementia and reported the results. Unlike many psychological tests, the proposed test does not require a specialized assistant and can be administered in a short time. The correlation coefficient between the total score of the SSWTRT and the MMSE score was *r* > 0.45, and in classification using the total score of the SSWTRT as the cutoff value, the classification performance for subjects with an MMSE score of 27 or less was specificity 0.74 and sensitivity 0.62 (AUC 0.7, cutoff value = 7.34).

Although the SSWTRT is a test designed to evaluate the state of the subject’s texture perception, based on the characteristics described above, improving the accuracy of classifying individuals with suspected cognitive decline (e.g., MMSE ≤27) is thought to be of practical value in dementia screening. In our previous report, we classified subjects based on the total score in the SSWTRT. However, as the tendency of responses to each question differs depending on the group classified according to the MMSE score, further improvement in classification performance can be expected by utilizing these individual differences. In this paper, we design a machine learning method that treats the answers to each question as individual elements and reports its performance.

There is a wide range of previous research into using machine learning to predict diseases based on patient health data. For example, it is known that diabetes and heart disease can be diagnosed with a high degree of accuracy by using health data such as a patient’s age, blood pressure, and lifestyle habits (Kopitar et al., 2020; Subramani et al., 2023). One example of previous research applying machine learning to the diagnosis of Alzheimer’s disease is a reported attempt at early diagnosis using MRI data (Pan et al., 2020).

The fact that decisions made by machine learning models are conducted in a black box has long been a significant problem in using these models for disease diagnosis. In particular, since misdiagnosis of a disease can harm the patient’s health, it is extremely important to understand the basis for the model’s judgment.

SHapley Additive exPlanations (SHAP), a method based on Shapley values from game theory, provides a quantitative explanation of how each feature contributes to a machine learning model’s predictions (Lundberg and Lee, 2017). This makes it easier to explain the model’s workings to stakeholders involved in the implementation of the model in society. In recent years, some studies have been reported that have attempted to examine the explanatory potential of SHAP models using patient physiological data (Yang et al., 2024); (Dharmarathne et al., 2024).

## 2 Sound Symbolic Words Texture Recognition Test

### 2.1 SSWRTR

In the SSWTRT, participants are shown a total of 12 close-up photographs of material surfaces (Supplementary Figure S1). For each image, as shown in Supplementary Figure S2, they are asked to select one of the eight SSW options that best represent the texture they perceive when touching the material. The details of the image stimuli used in the SSWTRT and the method for selecting SSWs are described in our previous study Kamohara et al. (2024) and shown in Supplementary Figures S1, S2, and Table 1.

**Table 1:** List of selected sound symbolic words (SSWs) and their corresponding meanings.

Sound symbolic word	Meaning
zara-zara	A texture and overall appearance that is coarse and has a strong roughness
tsuru-tsuru	A surface that is flat and glossy. A state of being smooth. Frequently used for hard materials such as boards or metals.
fuwa-fuwa	Softly swollen or puffed up in appearance
sara-sara	Lacking moisture or stickiness
gotsu-gotsu	Angular and hard in appearance. Not flexible or supple.
sube-sube	Smooth and pleasing to the touch; the condition of skin or hair is smooth
nuru-nuru	Slimy and slippery, causing discomfort, as if something mucous-like is clinging
deko-boko	A surface that is not flat, having bumps and indentations

Adapted from Kamohara et al. (2024), under CC BY 4.0 license.

The selected responses are then converted into scores by comparing them with the distribution of responses from a previous study conducted on a group of young, healthy participants. This scoring system is designed to assign higher scores to responses that align with those commonly chosen by young, healthy participants, while responses that deviate receive lower scores. Specifically, if a participant selects $x_{j}(1\leq j\leq 8)$ as the answer to the question $H_{i}$ (1≤i≤12) , the score is calculated using the following formula:


$$\text{Score}(x_{n},H_{i})=\frac{P(x_{n}|H_{i})}{\max_{1\leq j\leq 8}P(x_{j}|H_{i})}$$

Here, $P(x_{j}|H_{i})$ denotes the probability (the frequency obtained in an experiment on healthy subjects) that the healthy group will choose the answer $x_{j}$ for question $H_{i}$ . For example, selecting the most common response among healthy controls yields a score of 1, while selecting an option never chosen by them results in a score of 0.

**Figure 1:**
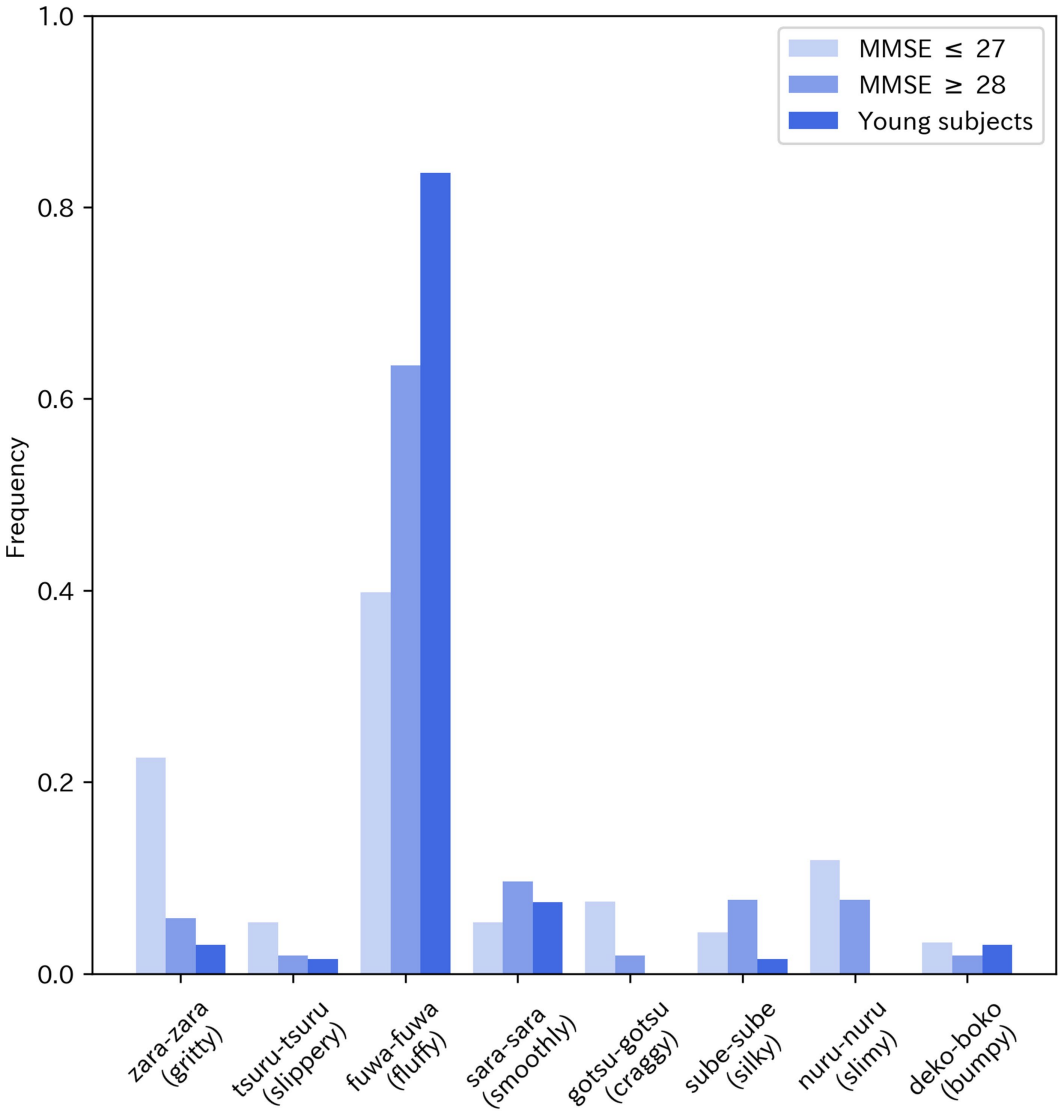
Distribution of subjects’ responses to Image 1. A shift from “fuwa-fuwa (fluffy)” to “zara-zara (gritty)” and “nuru-nuru (slimy)” was observed in the low-MMSE group.

Response patterns in the high-MMSE group closely matched those of healthy young controls, whereas the low-MMSE group more often endorsed options seldom chosen by controls. Figure 1 shows the distributions for Image 1 (fabric close-ups). Controls most frequently endorsed the sound-symbolic word *fuwa-fuwa* (“fluffy”; soft, puffy). In contrast, endorsements of *fuwa-fuwa* declined in the low-MMSE group, while selections of *zara-zara* (“gritty”; rough, snagging) and *nuru-nuru* (“slimy”; slippery, unpleasant) increased. Distributions of the remaining images are shown in Supplementary Figures 3(a–l).

### 2.2 Participant

A total of 233 subjects, including 102 subjects reported in our previous paper (patients who visited Juntendo University Hospital and Juntendo University Tokyo Koto Geriatric Medical Center from January to August 2023) and 131 patients who visited Juntendo University Hospital and Juntendo University Tokyo Koto Geriatric Medical Center from September 2023 to May 2024. The mean age of the participants was 77.06 years, with a SD of 7.25. Among the participants, 111 were male and 122 were female, and the patients were diagnosed with probable or definite iNPH by neurosurgeons and neurologists according to the Japanese iNPH guidelines (Dingemanse and Majid, 2012).

### 2.3 Ethical approvals

This study was approved by the Research Ethics Committee of Juntendo University, Tokyo, Japan (E22-0100). The preliminary experiment protocol was approved by the Research Ethics Committee of The University of Electro-Communications, Tokyo, Japan (#18026). The study adhered to the tenets of the Declaration of Helsinki, and written informed consent was obtained from all participants, including the preliminary experiment.

## 3 Classification using machine learning

In this section, we first describe the dataset used in the study. We then report the procedure for constructing the machine learning models and their classification performance. Finally, we present the results of the SHAP analysis, highlighting which variables the models considered most important for sample classification.

### 3.1 Data

**Figure 2:**
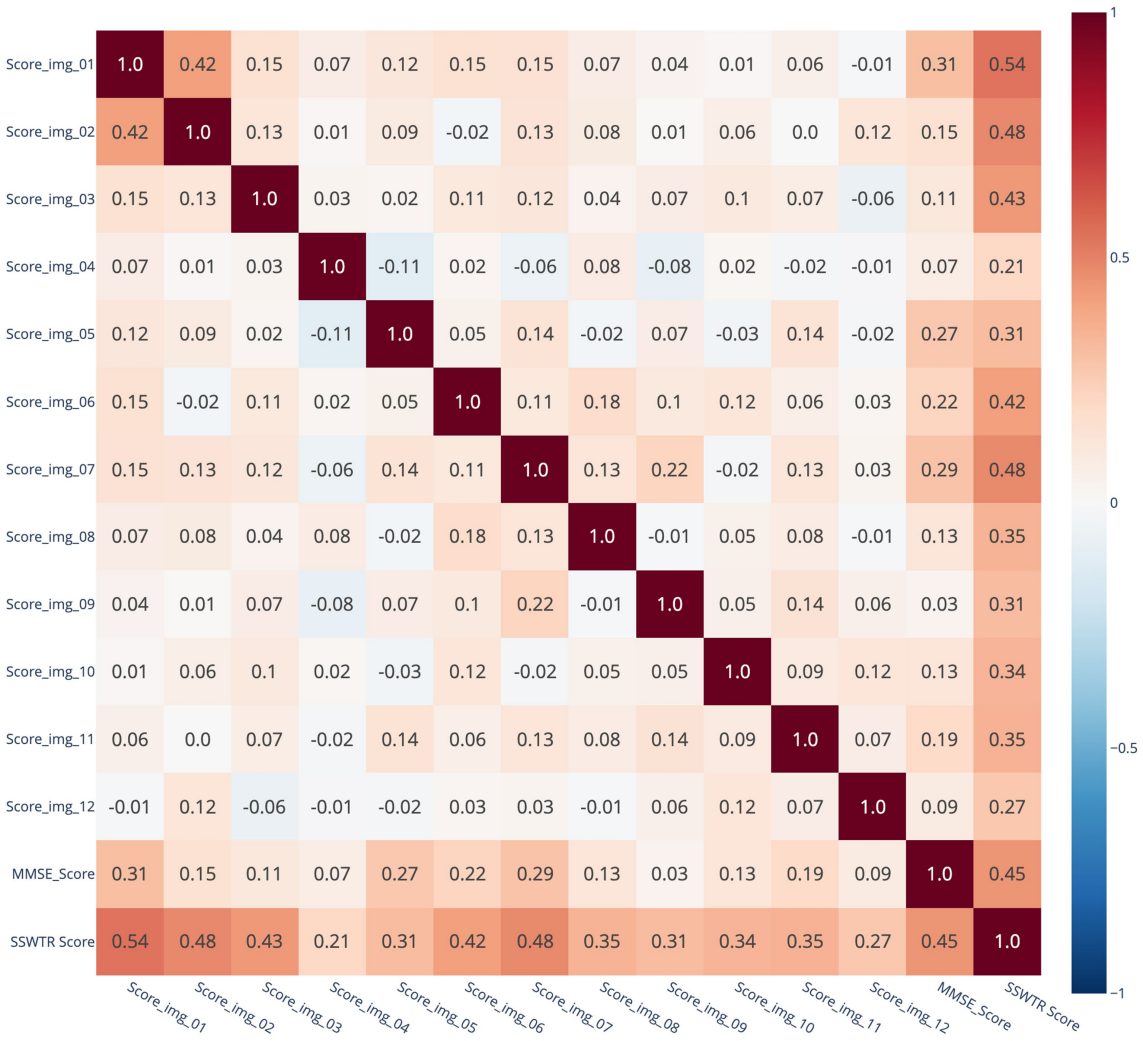
Correlation coefficient matrix between SSWTRT question scores, total score, and MMSE score.

**Figure 3:**
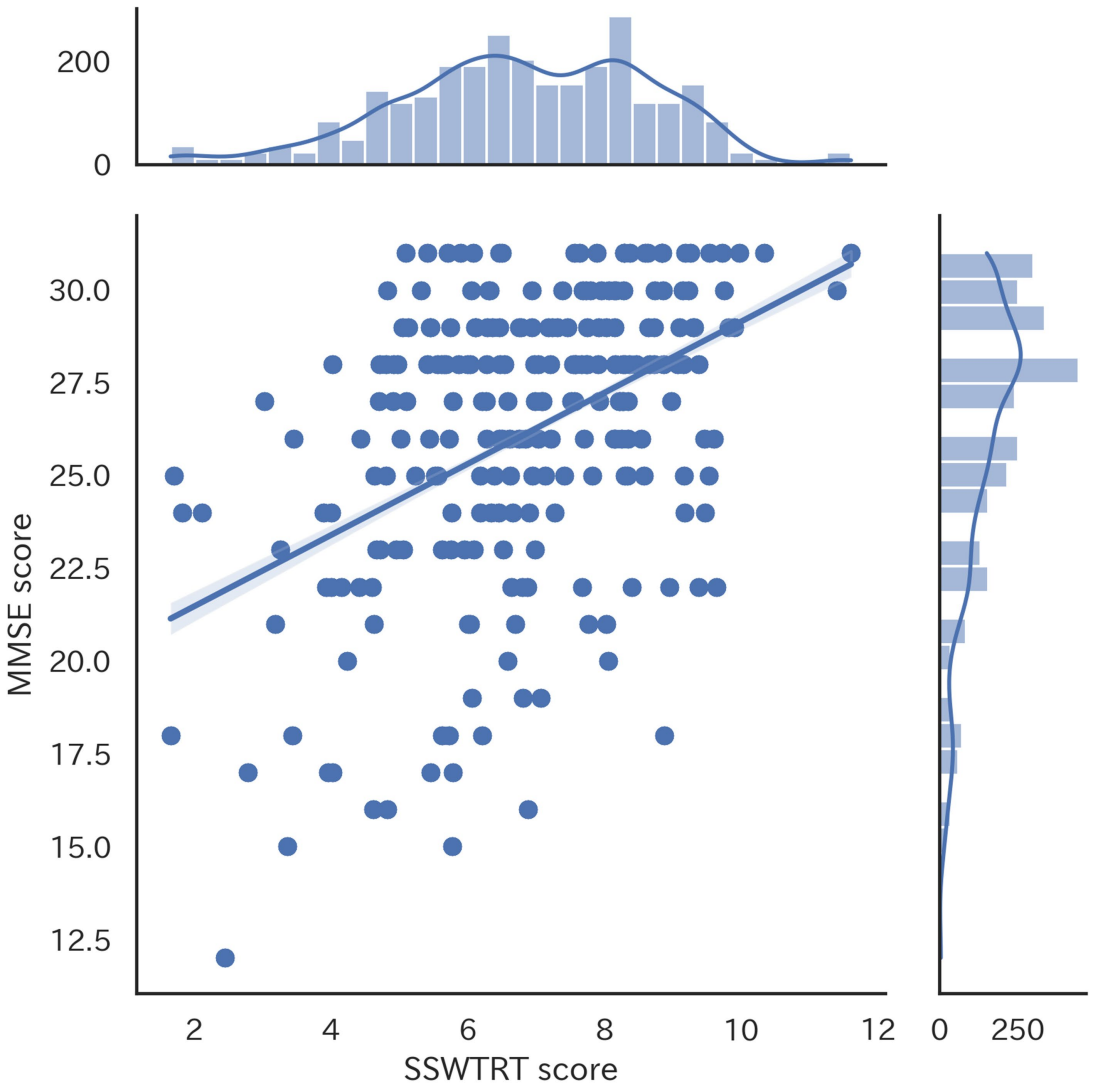
Scatter plot shows the correlation between SSWTRT total scores and MMSE scores.

This section provides an overview of the data used for machine learning. As described above, the dataset includes a total of 233 samples. Each record contains 14 attributes: the SSWTRT score calculated from each subject’s responses to the 12 images, the total score for the 12 questions on the SSWTRT, and the subject’s MMSE score. Figure 2 shows the correlation coefficient matrix between the scores for the 12 questions in the SSWTRT, the total score of the SSWTRT, and the MMSE score. The correlation coefficient between the total score of the 12 questions in the SSWTRT and the MMSE score was 0.45. To better understand the correlation between the SSWTRT total score and the MMSE score, a scatter plot is shown in Figure 3.

**Figure 4:**
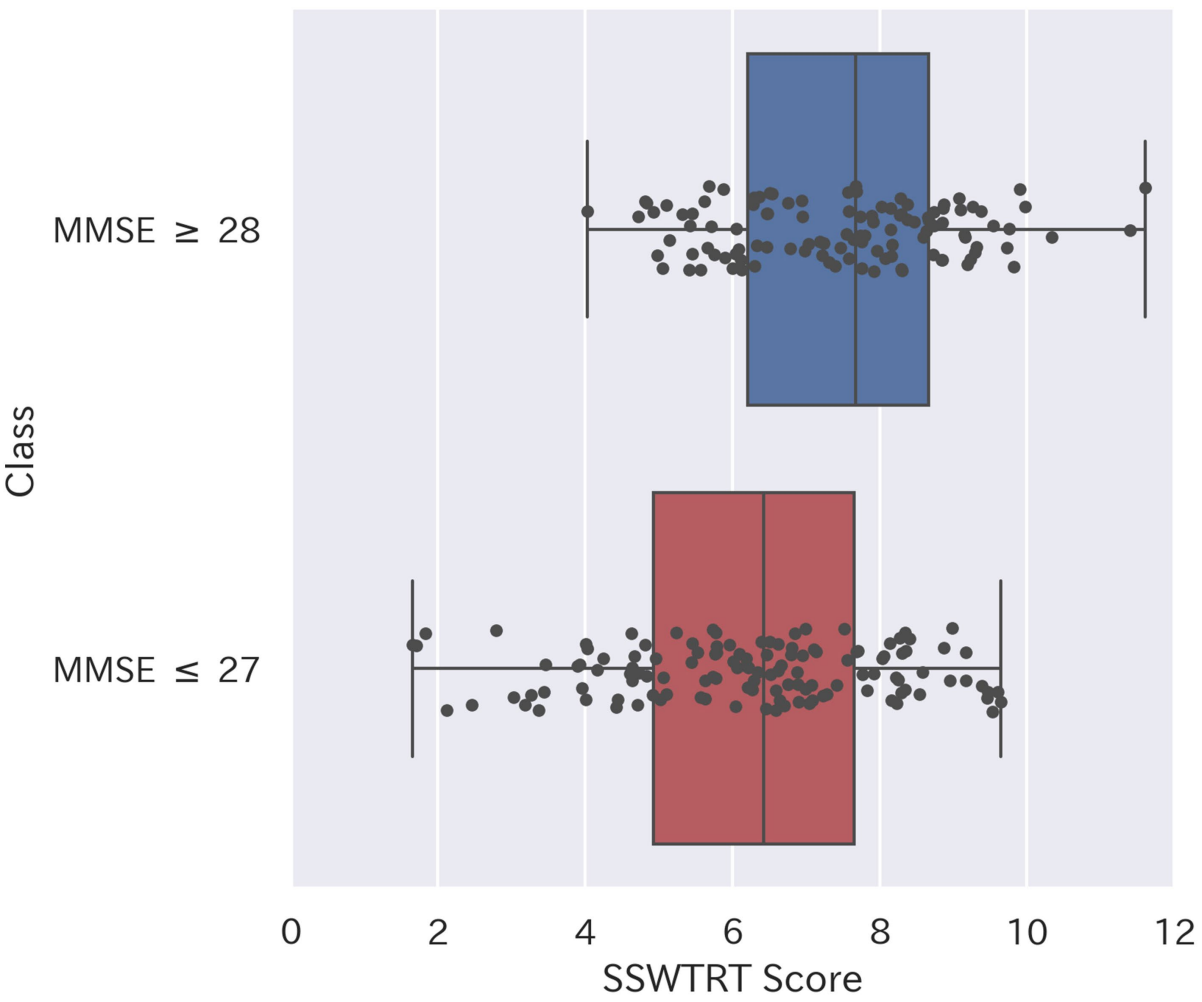
Boxplot compares SSWTRT scores between MMSE ≤27 and MMSE ≥28 groups (*p* < 0.001).

Figure 4 shows the box plot of the SSWTRT scores divided into two groups based on the MMSE score. In this study, a cutoff indicating cognitive decline was set based on previous research on the criteria for diagnosing MCI, and participants were divided into two groups (Cuoco et al., 2025; Zhang et al., 2021).

A significant difference (<0.001**, *t*-test) was confirmed between the mean scores of the group with an MMSE score of 27 or less, which suggests cognitive decline, and the group with an MMSE score of 28 or more. The number of subjects in the group with an MMSE score of 27 or less was 111, and the number of subjects in the group with an MMSE score of 28 or more was 126. The original dataset also included results from other cognitive assessments, such as the FAB and RAVLT, as well as demographic information such as participant age. However, since the aim of this study was to evaluate the classification performance of the SSWTRT as an independent screening tool requiring minimal time and resources, we only utilized data on participants’ age, education levels, and responses to each question on the SSWTRT. Comparison of the SSWTRT with the results of other mental tests was provided in our previous paper (Kamohara et al., 2024).

### 3.2 Model build and performance evaluation

Using the differences in response trends between groups in the SSWTRT discussed in the previous section, we designed a machine learning model to classify subjects with an MMSE score of 27 or less, suspected of having mild cognitive impairment. In addition to the scores of each subject’s responses to the 12 questions, we included age and years of education as input features, with missing values in years of education imputed by the mean. We applied three machine learning methods: *K*-nearest neighbors (KNNs), random forest classifier (RFC), and support vector machine (SVM). For RFC and SVM, we evaluated models both with and without applying balance control techniques (Bach et al., 2019; Chawla et al., 2002; Lin et al., 2020) designed to improve performance on minority classes in imbalanced data. Model performance was evaluated using 5-fold cross-validation, based on accuracy, precision, recall, *F*1 score, and AUC, selecting the model that achieved the highest *F*1 score. For hyperparameter tuning with 5-fold cross-validation, the dataset was divided into five folds, and for each candidate set of hyperparameters, the model was repeatedly trained on four folds and evaluated on the remaining one. The five evaluation scores obtained were averaged, and the hyperparameters yielding the best mean performance were selected. Finally, the model was retrained on the entire dataset using the optimal hyperparameters to obtain the final model.

### 3.3 Result

**Figure 5:**
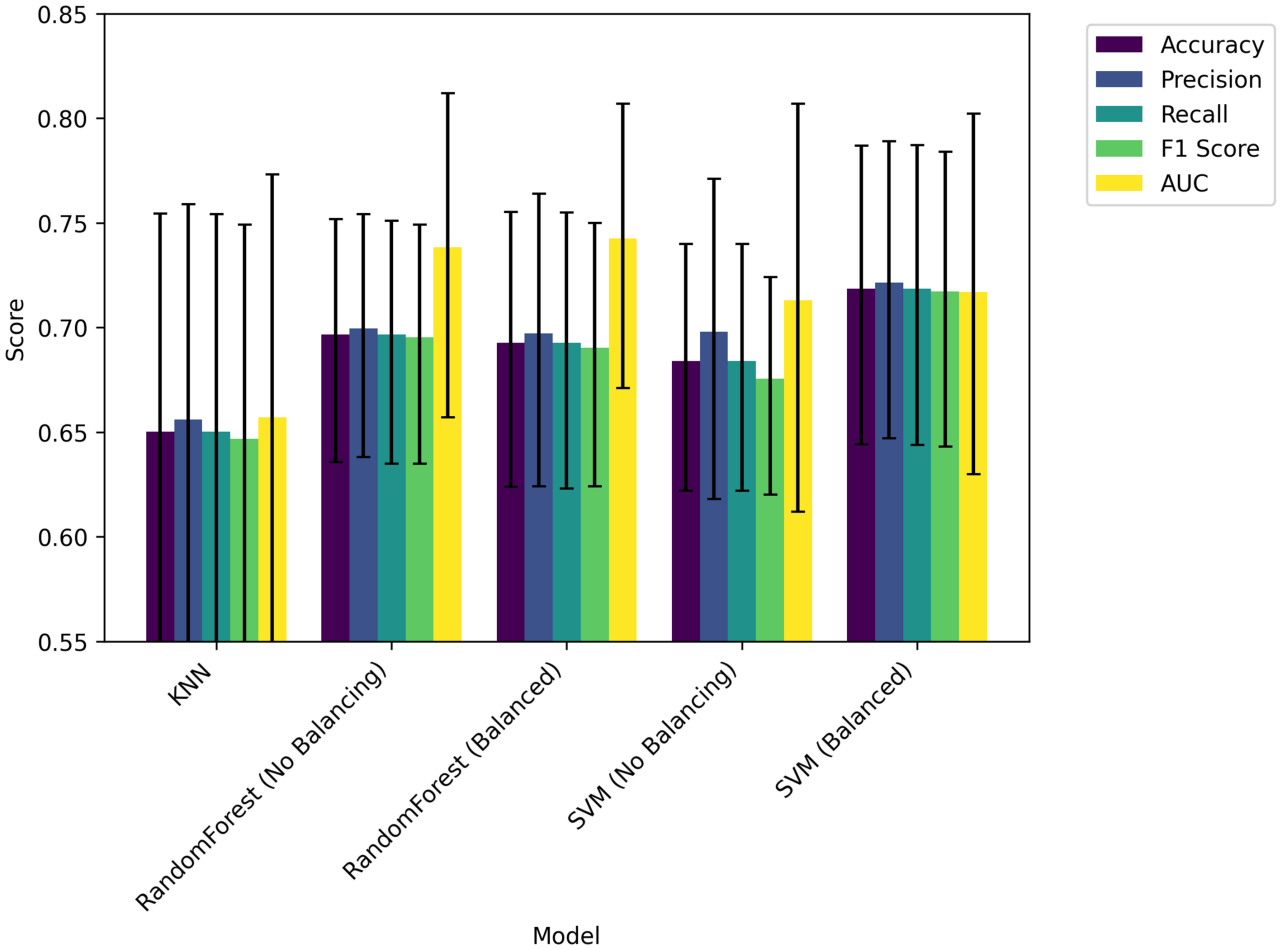
Performance comparison among five machine learning models based on accuracy, precision, recall, *F*1 score, and AUC.

**Figure 6:**
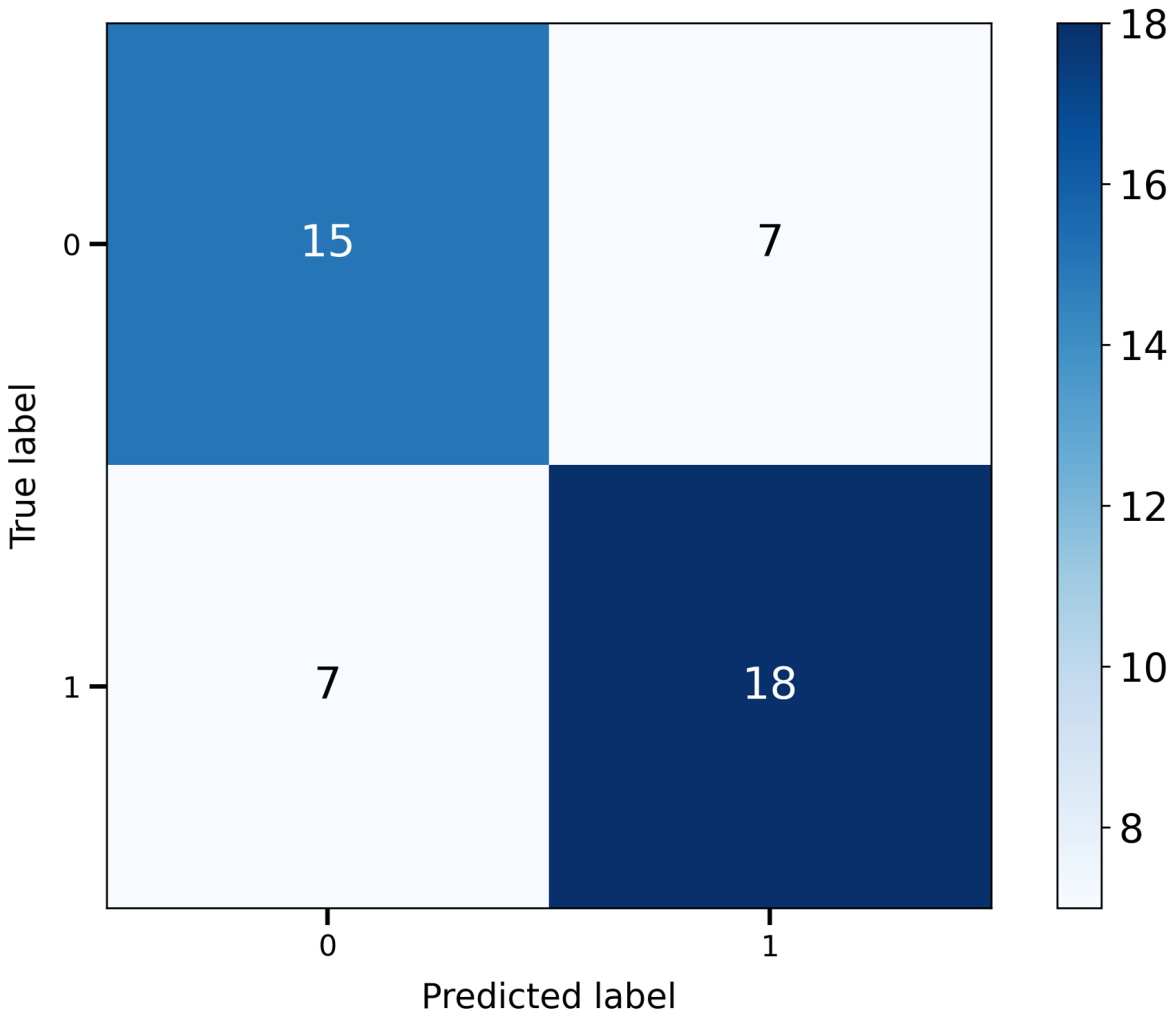
Confusion matrix for SVM classification results from the validation dataset.

For each method, we optimized each model using the hyperparameters in the ranges shown in Table 2 and compared the performance of each model on five indices: accuracy, precision, recall, *F*1 score, and AUC (ROC-AUC score) (Table 3; Figure 5). The best performance, except for AUC, was achieved using SVM with SMOTE. The best model had accuracy = 0.71, precision = 0.72, recall = 0.72, *F*1 score = 0.72, and AUC = 0.72. The confusion matrix for the classification results for 47 validation set samples (20% of 233 total participants) using this model is shown in Figure 6.

**Table 2:** Optimized hyperparameters.

Classifier	Hyperparameter	Values	Description
KNN	*n*_neighbors	3, 5, 7, 9	Number of neighbors to consider when classifying a data point.
	weights	Uniform, distance	Weighting method for neighbors; ‘distance’ assigns greater weight to closer neighbors.
	*p*	Manhattan, euclidean	Distance metric: *p* = 1 (Manhattan), *p* = 2 (Euclidean).
Random forest	*n*_estimators	50, 100, 200	Number of trees in the forest.
	max_depth	None, 5, 10	Maximum depth of each tree; controls model complexity.
	min_samples_split	2, 5, 10	Minimum number of samples required to split an internal node.
SVM	C	0.1, 1, 10	Regularization parameter: balances margin size and misclassification.
	kernel	linear, RBF	Kernel type used to transform the input data space.

**Table 3:** Performance evaluation of models.

Classifier	Best Params (summary)	Accuracy	Precision	Recall	F1 score	AUC
KNN	*n*_neighbors: 5,	0.65	0.66	0.65	0.65	0.66
	p: Manhattan,					
	weights: uniform					
RFC (no balancing)	*n*_estimators: 50,	0.70	0.70	0.69	0.70	0.74
	max_depth: 10,					
	split: 10					
RFC (balanced)	*n*_estimators: 100,	0.69	0.70	0.69	0.69	0.74
	max_depth: None,					
	split: 10					
SVM (no balancing)	C: 10,	0.68	0.70	0.68	0.68	0.71
	kernel: RBF					
SVM (balanced)	C: 10,	0.71	0.72	0.72	0.72	0.72
	kernel: RBF					

**Figure 7:**
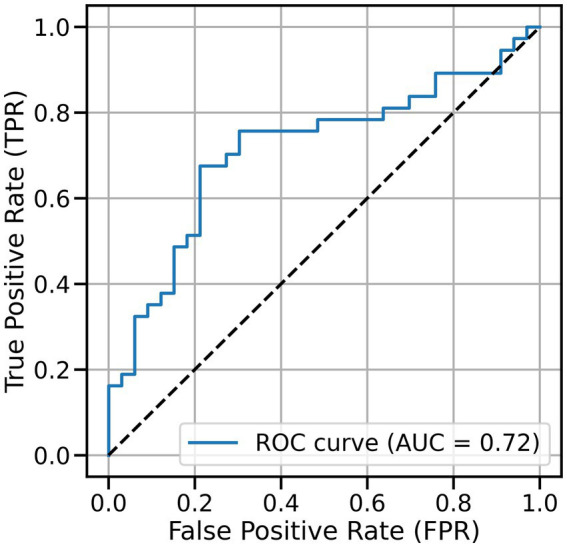
Receiver operating characteristic (ROC) analysis of the SVM classifier, where decision scores were obtained from the signed distances of samples to the separating hyperplane.

Using the best SVM model, we performed ROC analysis using the distance between each sample and the hyperplane (Figure 7). The TPR increased to 0.7 or higher when the FPR was approximately 0.3, which is a balanced operating point that maintains relatively high sensitivity while also ensuring a certain level of specificity. The optimal threshold for application will vary slightly depending on the combination with other screening tests and will need to be adjusted depending on whether sensitivity or specificity is prioritized.

### 3.4 Model explanations

In this section, we describe the results of investigating the decision-making process of the machine learning model developed in this study using SHAP. Using SHAP, we can quantitatively evaluate the degree of influence of each input feature on prediction, which not only helps us understand the model’s decision-making process but also allows us to evaluate whether the decision is reliable. Model explainability helps to gain understanding from various stakeholders when implementing the developed model in society, and contributes to continuous model development, data collection, and improvement of experimental conditions.

**Figure 8:**
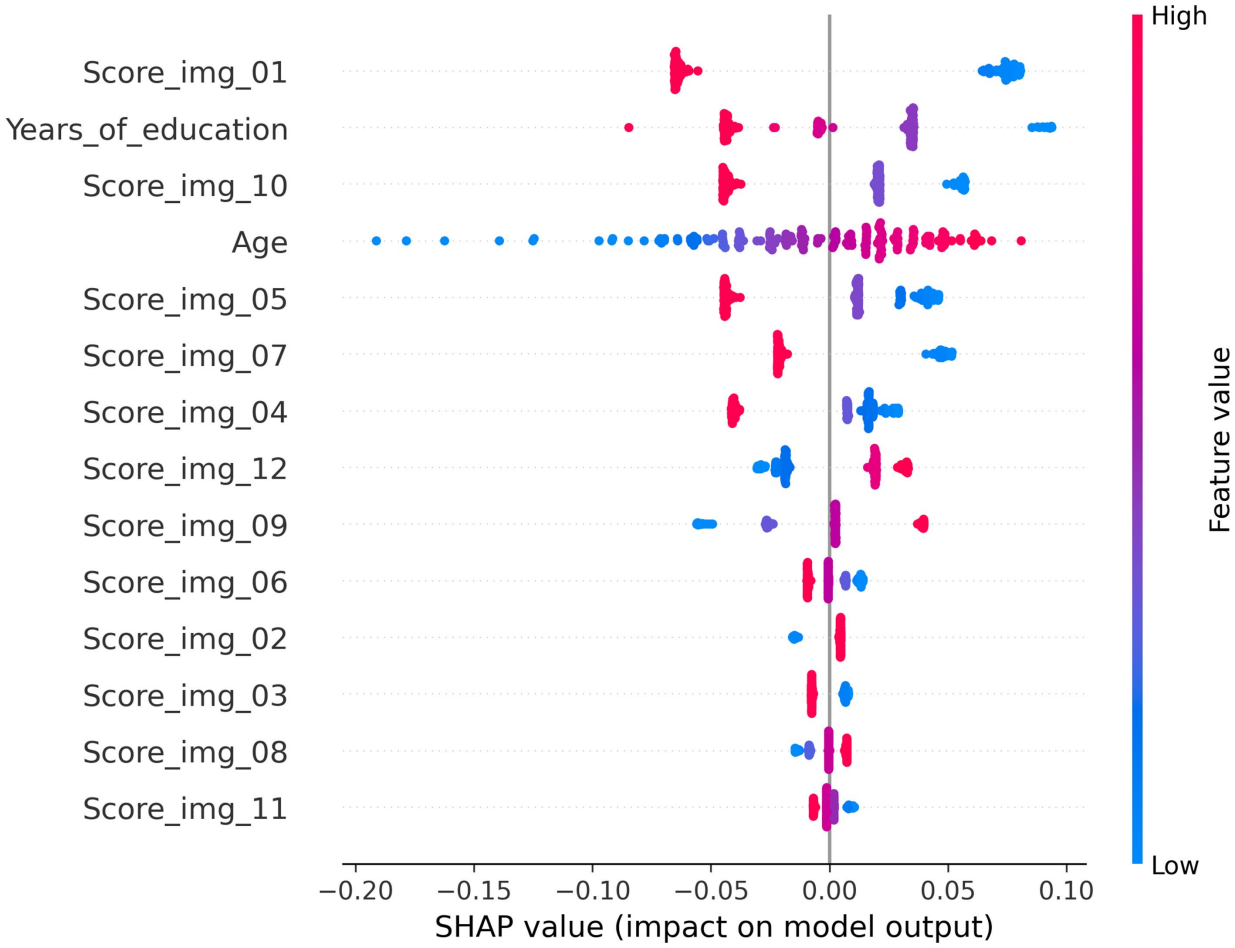
SHAP summary plot shows the importance and directionality of each feature (image score) in the classification model.

The analysis using SHAP in this section was performed on the SVM model that achieved the highest classification performance in the comparison of indicators in the previous section. Figure 8 presents the SHAP summary plot, in which the features on the vertical axis are arranged in descending order of their impact on the model output. In the SHAP summary plot, the horizontal axis represents the feature importance (SHAP values), with red points indicating higher feature values and blue points indicating lower values. The horizontal spread reflects variability in the impact of each feature.

**Figure 9:**
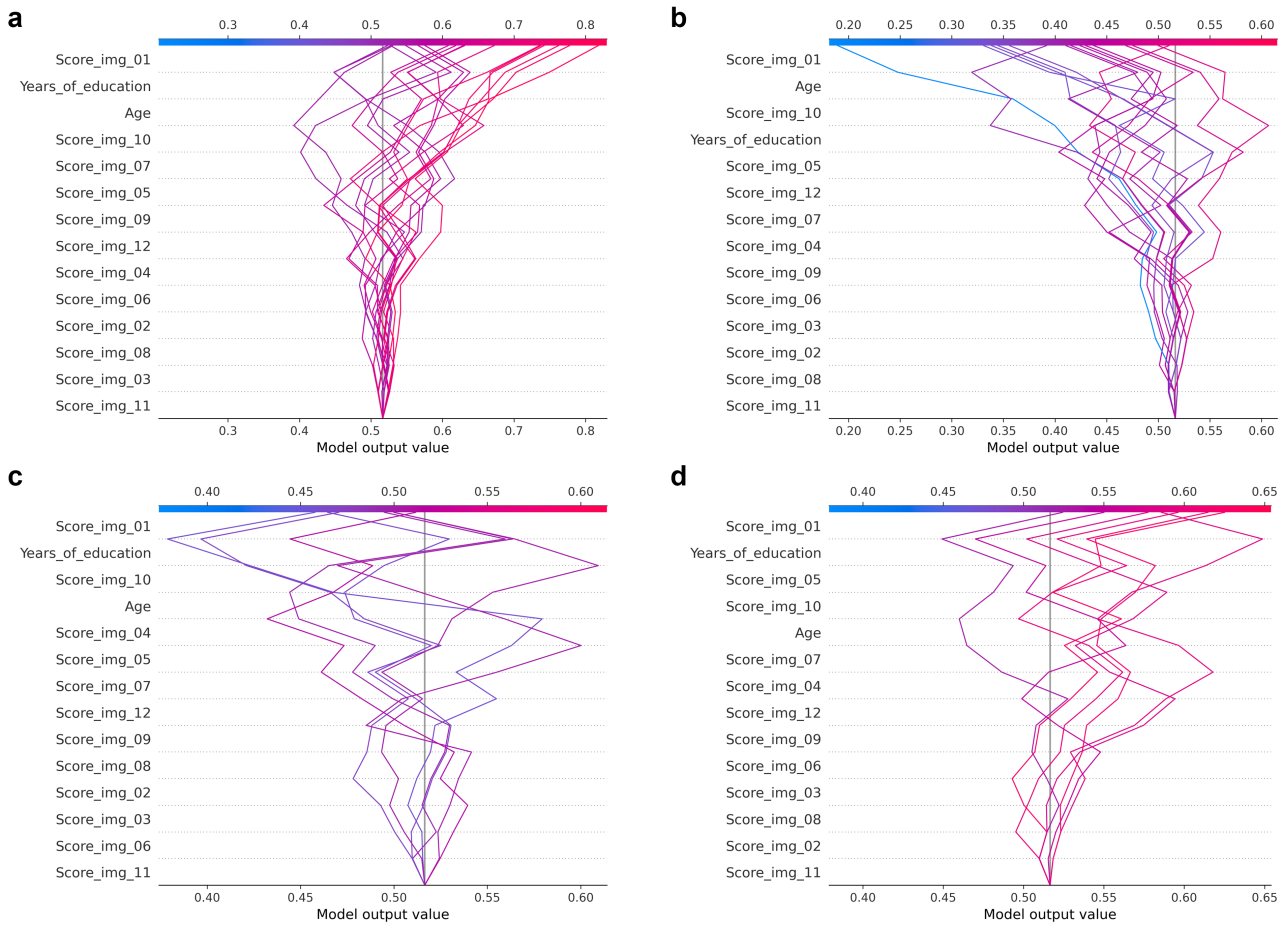
SHAP decision plots generated from the Random Forest classifier. The plots show feature contributions for all test samples stratified by classification outcome: **(a)** correctly classified samples of Class 1, **(b)** correctly classified samples of Class 0, **(c)** misclassified samples of Class 1, and **(d)** misclassified samples of Class 0.

As shown in the figure, when participants were classified into MMSE score groups using the SSWTRT question scores, the most important question was identified as Image 01. Notably, the importance of responses to Image 01 exceeded that of participants’ years of education or age. The SHAP values transition from red to blue from left to right, indicating that higher Image 01 scores exert a stronger effect in pushing the classifier’s output toward Class 0 (i.e., the group with MMSE ≥28). Subsequently, years of education, Image 10, age, Image 05, and Image 07 followed in descending order of variable influence. For these image-related items, the model appeared to learn that higher response accuracy increased the likelihood of classification into the cognitively normal group. In contrast, for items such as Images 12 and 09, higher response accuracy was associated with a greater likelihood of classification into Class 1. As shown in the correlation coefficient matrix in the previous section, Images 01 and 05 exhibited relatively high correlations with MMSE scores, whereas Images 09 and 12 demonstrated low or even negative correlations. These findings suggest that such questions may not function effectively in the classification process. However, the contribution of these variables (Images 09 and 12) to the classification was low. The SSWTRT question scores were originally derived from data obtained from young adults presumed to be free of cognitive impairment. Therefore, the decision-making process of the machine learning model—linking higher scores on certain questions with a greater likelihood of cognitive decline—deviates from the intended scoring design and requires further refinement. Approaches for addressing this issue will be discussed in the following section. Figures 9a–d illustrates decision plots that show how the classifier evaluated the features of individual samples in the test set and produced classifications. SHAP decision plots are visualization tools that reveal how a machine learning model generates its predictions. By displaying the cumulative contributions of each feature alongside the final output, they enable detailed analysis of the decision-making process for each sample. Figures 9a,b present decision plots for correctly classified samples, whereas Figures 9c,d depict those for misclassified samples. Comparisons between these plots provide insights into potential improvements for both the SSWTRT test and the classifier.

In the decision plots of misclassified participants, the classifier’s outputs tend to cluster approximately 0.5 across many samples, in contrast to the patterns observed in correctly classified cases. For example, in the misclassified class 1 sample shown in Figure 9c, all outputs fall within the narrow range of 0.45–0.52. Moreover, in many misclassified samples, the decision paths of Image 01 and years of education intersect, suggesting the need for refinement to better detect such specific patterns.

## 4 Discussion

We demonstrated that utilizing responses to individual questions in the SSWTRT improved classification accuracy into MMSE-based groups that indicate a possible risk of cognitive decline. The ROC analysis demonstrated moderate discriminative ability (AUC = 0.72), with sensitivity and specificity approximately 0.72 at the optimal threshold, suggesting potential utility as a supplementary measure rather than a standalone diagnostic test. Importantly, the SSWTRT can be administered without requiring specialized personnel, thereby reducing the burden on examiners, while patients may experience less anxiety or embarrassment compared to conventional cognitive tests. This ease of administration, combined with the possibility of implementation on a single device such as a tablet, makes the test potentially more accessible and cost-effective.

Model interpretability analysis using SHAP indicated that some images may play an important role in classification, while others may not. Since a higher SSWTRT total score is closer to the texture perception of healthy subjects, each score is expected to have a positive effect on predicting the MMSE group, but some questions showed the opposite effect. This may be due to perceptual misjudgment caused by an age-related decline in sensory function rather than cognitive function itself, introducing noise into the prediction process. Considering previously reported visual characteristics in dementia patients, such as reduced contrast sensitivity (Risacher et al., 2013; Hutton et al., 1993), future studies should increase the number of test images and analyze the relationship between image features and classification performance.

The analysis of the SHAP decision plot showed that many of the misclassified samples had classifier outputs concentrated at approximately 0.5, suggesting that they were samples for which the classifier was “unconfident.” In actual screening sites, it may be effective to present the confidence level of the output and introduce a multi-stage evaluation method that combines other tests as necessary.

The SSWTRT presents each question with eight response options, and scoring is computed using the formula detailed in Section 2. This mechanism assigns higher scores to responses that align more closely with those of healthy individuals, while deviations result in lower scores. In this study, a machine learning model was constructed using the 12 individual question scores together with age and years of education as input features. Beyond numerical scoring, leveraging the categorical nature of selected response options as features could provide additional insights. While this approach would lead to a sparser feature space requiring a larger dataset, it may offer a potential solution to the observed classification limitations.

Future research should focus on collecting a larger dataset, evaluating alternative feature representations, and analyzing variations in feature importance across different images. Additionally, optimizing image selection to enhance classification efficacy could further improve the performance of the SSWTRT and strengthen its role as a practical and accessible screening tool for cognitive decline.

## 5 Limitations

This study has several limitations. First, the participants consisted exclusively of patients diagnosed with idiopathic normal pressure hydrocephalus (iNPH). Therefore, the findings and the proposed model should be interpreted with caution, as their generalizability to other populations—such as patients with Alzheimer’s disease, individuals with mild cognitive impairment, or cognitively healthy elderly adults—remains highly uncertain and requires further validation. Second, some SSWTRT scores may be influenced by age-related sensory decline (e.g., visual or tactile), not purely cognitive deterioration. This could introduce noise or reverse the intended relationship between score and cognitive state. Finally, since the SSWTRT utilizes Japanese sound symbolic words (SSWs), the test’s cultural and linguistic specificity limits its immediate applicability to non-Japanese-speaking populations. Future studies should explore language-independent representations of texture recognition.

## 6 Summary

In this study, we analyzed both the previously reported results of the SSWTRT and newly collected experimental data, developing multiple machine learning models to predict participants’ MMSE score groups (≥28 or ≤27) using individual question scores, years of education, and age as explanatory variables. Among these, the best-performing classifier achieved an accuracy of 0.71, a precision of 0.72, a recall of 0.72, an F1 score of 0.72, and an AUC of 0.72. These results indicate that classification based on individual SSWTRT question scores provides higher accuracy compared to conventional models that rely solely on the total test score. This finding highlights the potential utility of integrating SSWTRT with machine learning techniques for screening individuals at risk of cognitive decline, as defined by MMSE scores, rather than focusing only on overall performance.

Model explainability analysis further revealed that certain image-based questions were more informative for classification than participants’ demographic factors, such as age and years of education. Conversely, the analysis suggested that some items may contribute to classification in a direction opposite to that intended by the original test design. These insights provide an important foundation for refining both the test itself and the machine learning models built upon it.

Future studies will focus on examining the characteristics of items that exert strong influence versus those with minimal impact on classification, to further improve predictive accuracy. Notably, the developed model is lightweight and computationally efficient, indicating the potential for real-time inference with minimal resource requirements. Taken together, these findings imply that the proposed framework may have practical value as a scalable screening tool for individuals at risk of cognitive decline, although further refinement and validation are needed to establish its clinical applicability.

## Data Availability

The raw data supporting the conclusions of this article will be made available by the authors, without undue reservation.
