# Correction: Machine learning-based detection of cognitive decline using SSWTRT: classification performance and decision analysis

**DOI:** 10.3389/frai.2025.1764066

**Published:** 2026-01-08

**Authors:** Yuji Nozaki, Chihiro Kamohara, Ryota Abe, Taiki Ieda, Madoka Nakajima, Maki Sakamoto

**Affiliations:** 1 Department of Informatics,Graduate School of Informatics and Engineering, The University of Electro-Communications, Chofu, Japan; 2 Research Institute for Diseases of Old Age, Juntendo University School of Medicine, Tokyo, Japan; 3 Department of Neurosurgery, Juntendo University School of Medicine, Tokyo, Japan

**Keywords:** sound symbolic words, texture recognition, dementia, neuropsychological tests, machine learning, SHAP

In the original article, there was a mistake in Figure 9 as published.

The subpanels **(a)** and **(d)** were erroneously interchanged causing the displayed order of panels (a, b, c, d) to differ from the intended structure described in the article text.

The corrected figure image is provided below.

**Figure 9:**
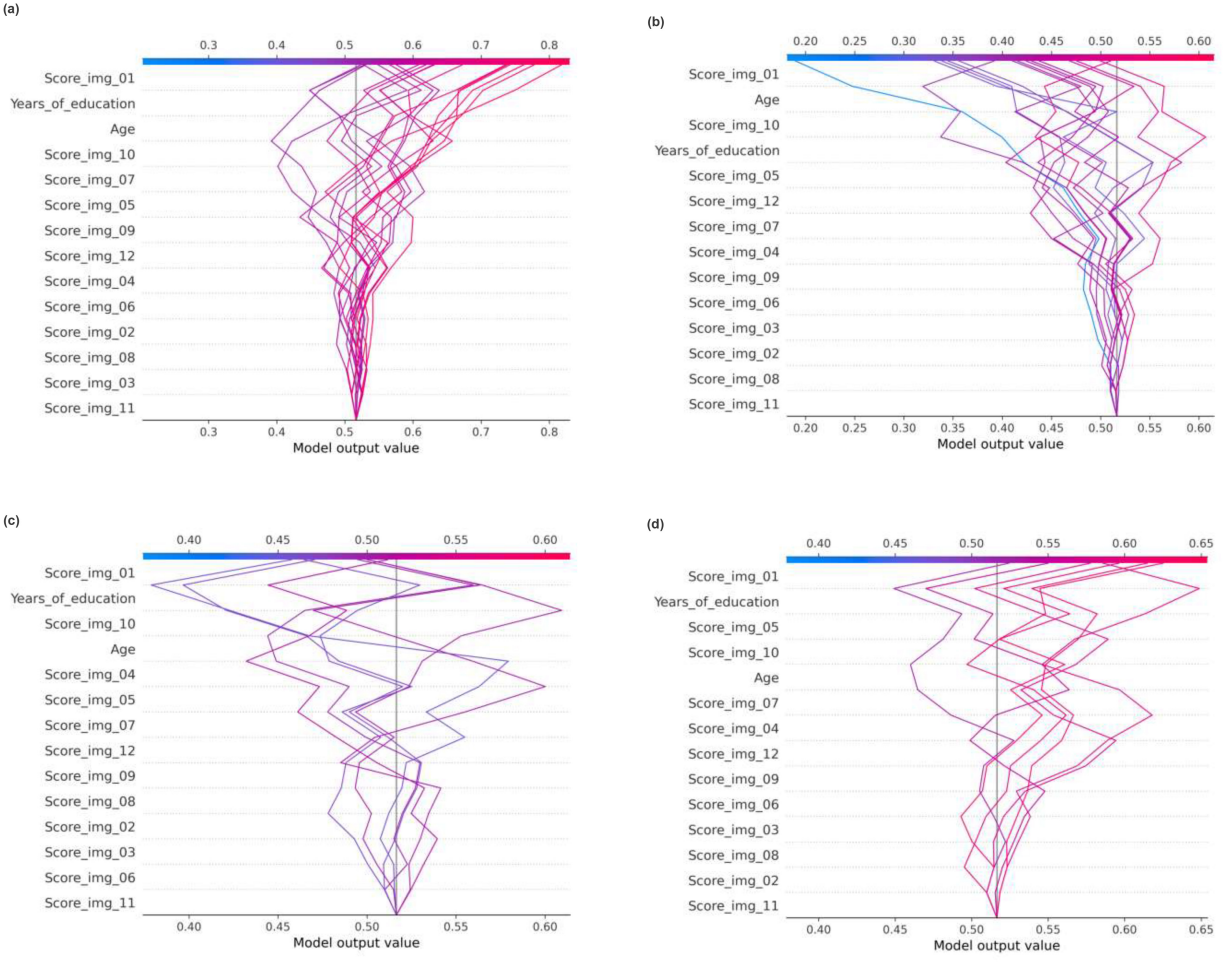
SHAP decision plots generated from the Random Forest classifier. The plots show feature contributions for all test samples stratified by classification outcome: **(a)** correctly classified samples of Class 1, **(b)** correctly classified samples of Class 0, **(c)** misclassified samples of Class 1, and **(d)** misclassified samples of Class 0.

The original article has been updated.

