# Supplementary figures and images for: Machine learning-based detection of cognitive decline using SSWTRT: classification performance and decision analysis
Source: Front Artif Intell. 2025 Oct 29;8:1689182. doi: 10.3389/frai.2025.1689182 (PMC12605398; doi:10.3389/frai.2025.1689182)

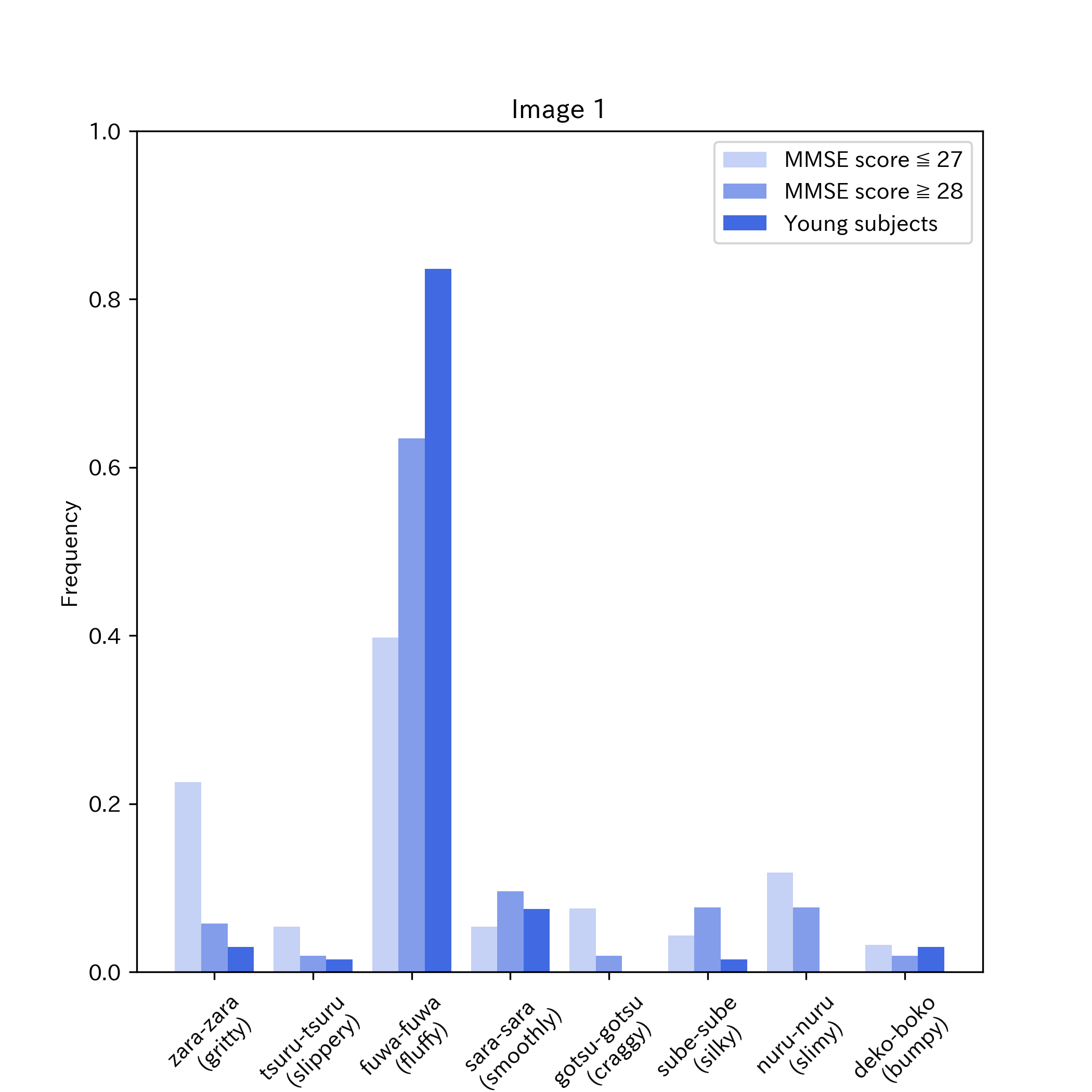

Supplement: Supplementary file 1 [file Supplementary_file_1.zip › S/FigS3a.jpg]

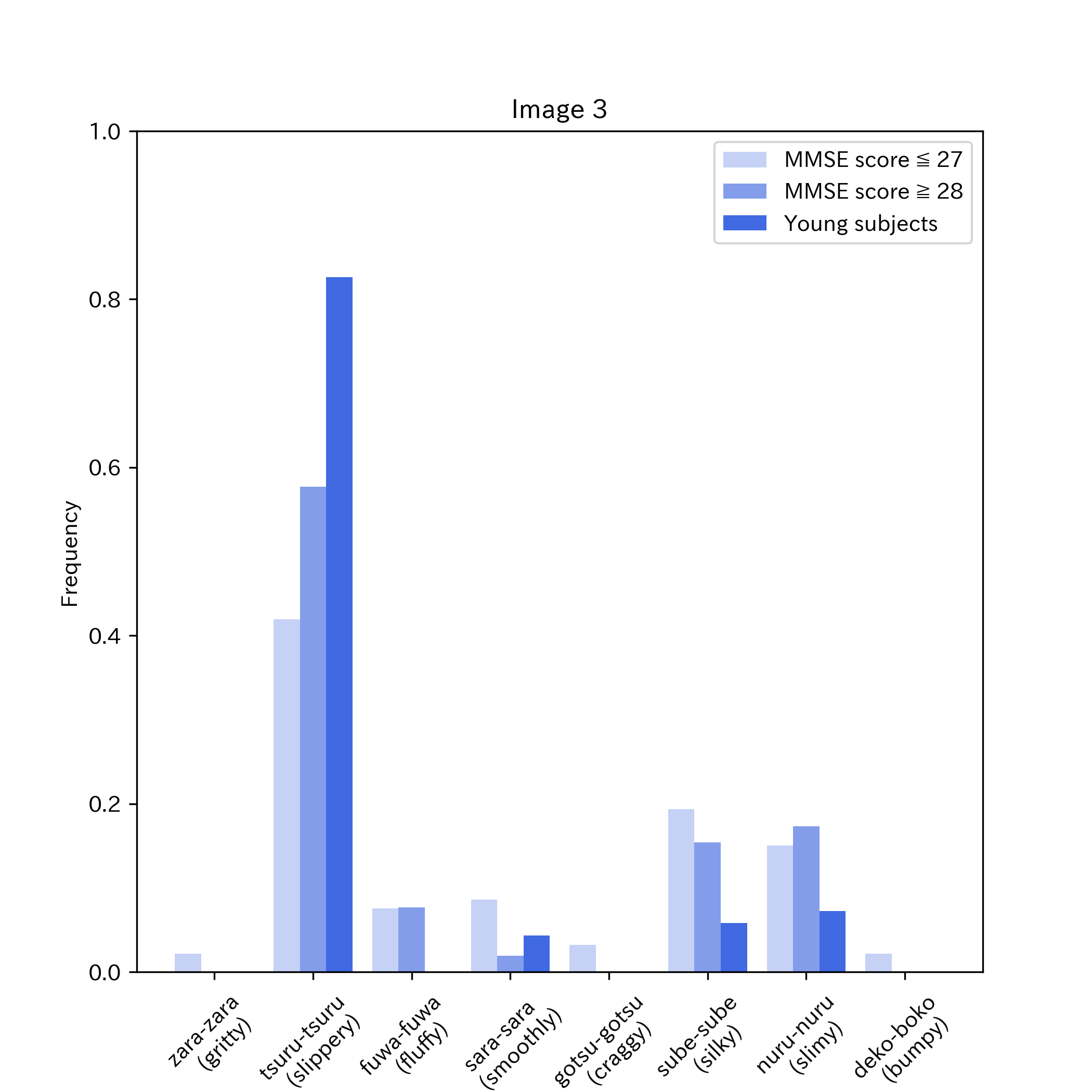

Supplement: Supplementary file 1 [file Supplementary_file_1.zip › S/FigS3c.jpg]

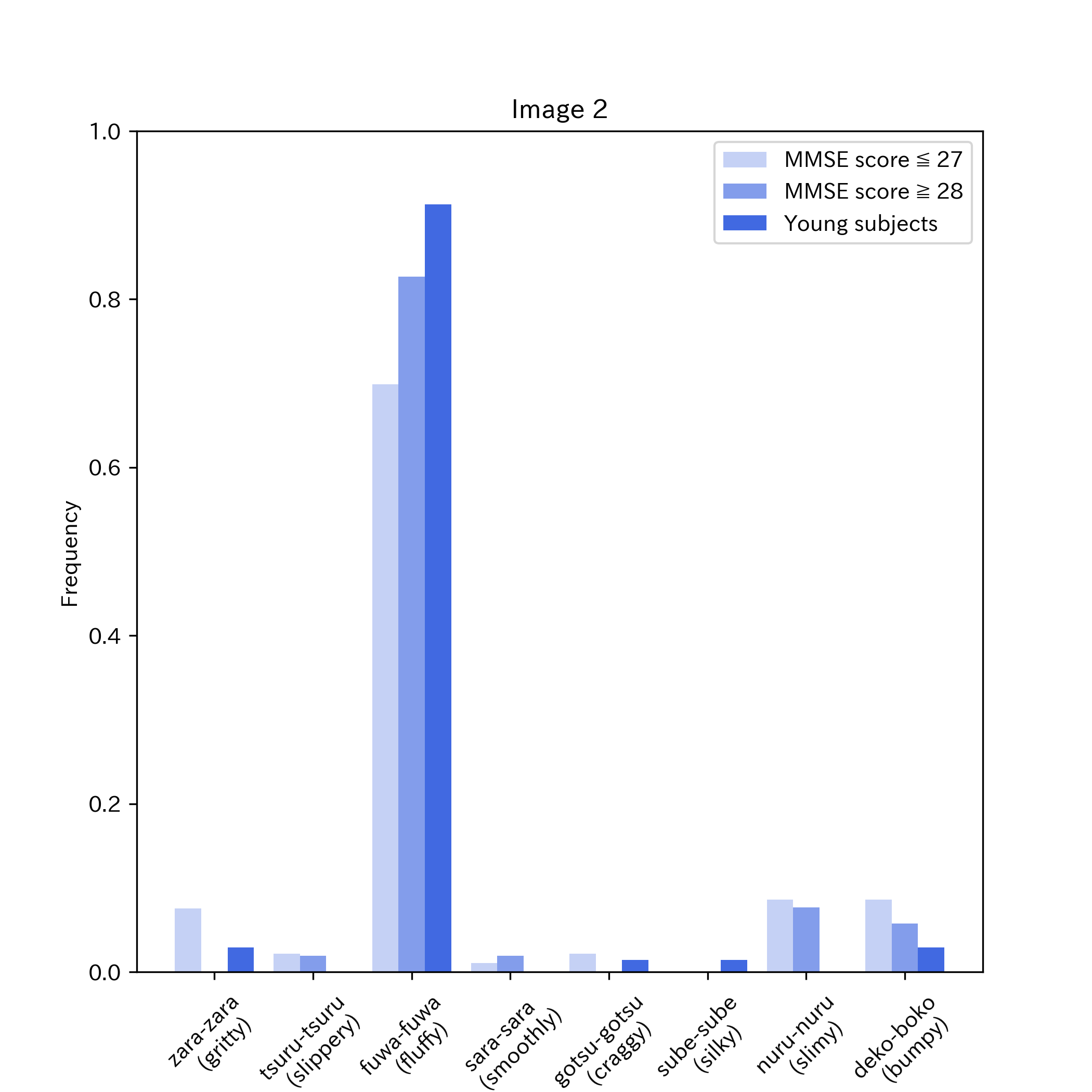

Supplement: Supplementary file 1 [file Supplementary_file_1.zip › S/FigS3b.jpg]

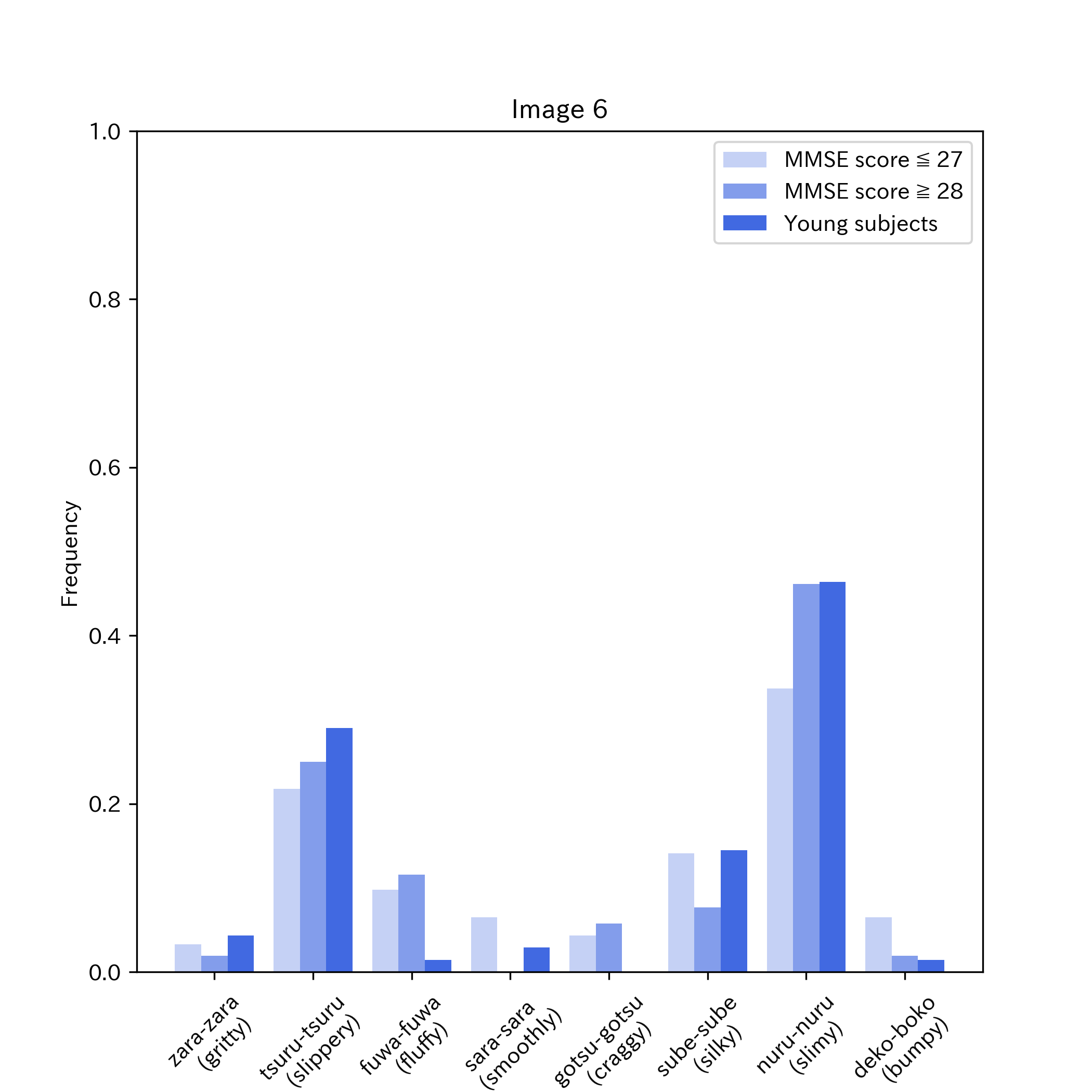

Supplement: Supplementary file 1 [file Supplementary_file_1.zip › S/FigS3f.jpg]

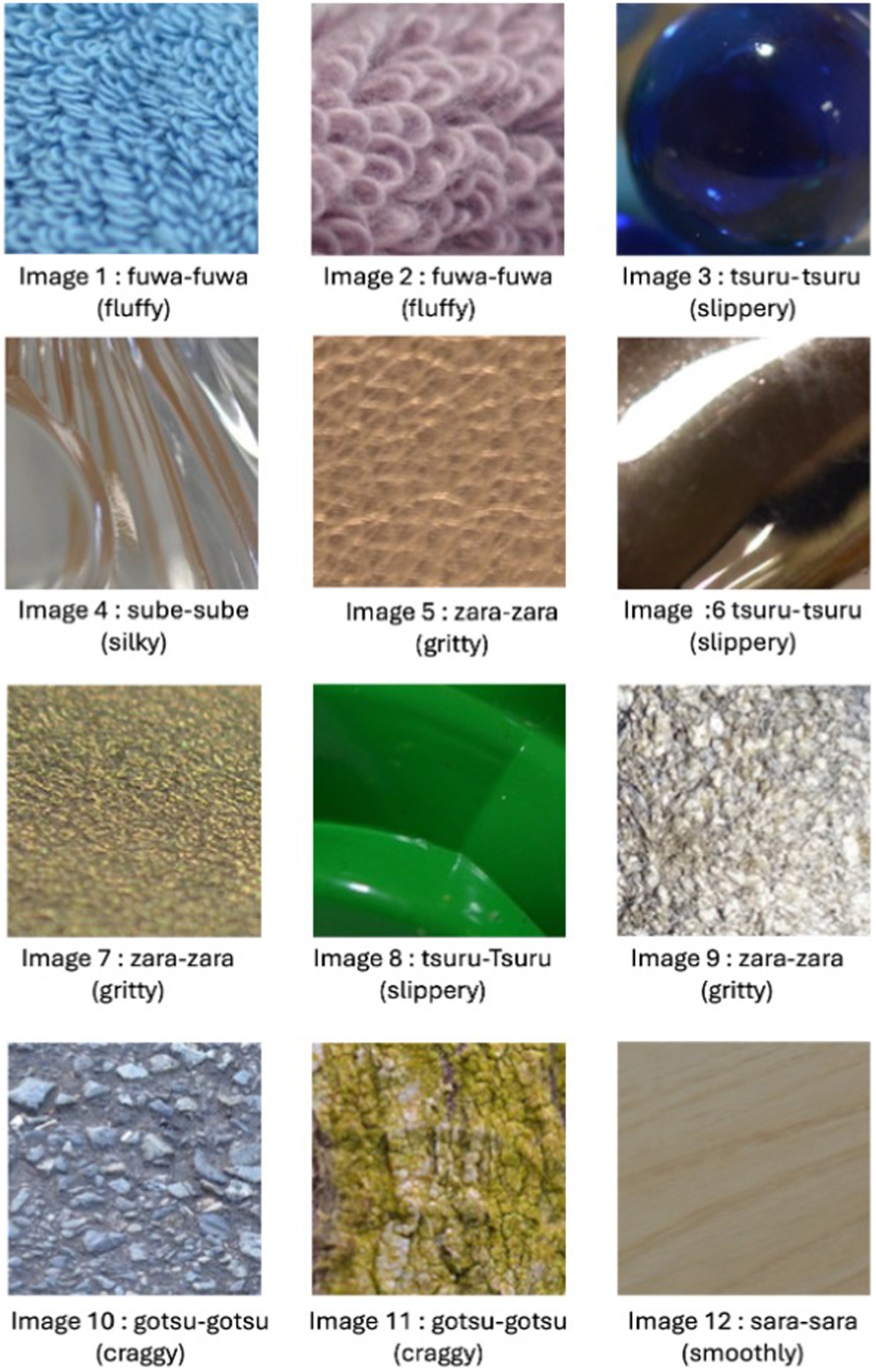

Supplement: Supplementary file 1 [file Supplementary_file_1.zip › S/FigS1.jpg]

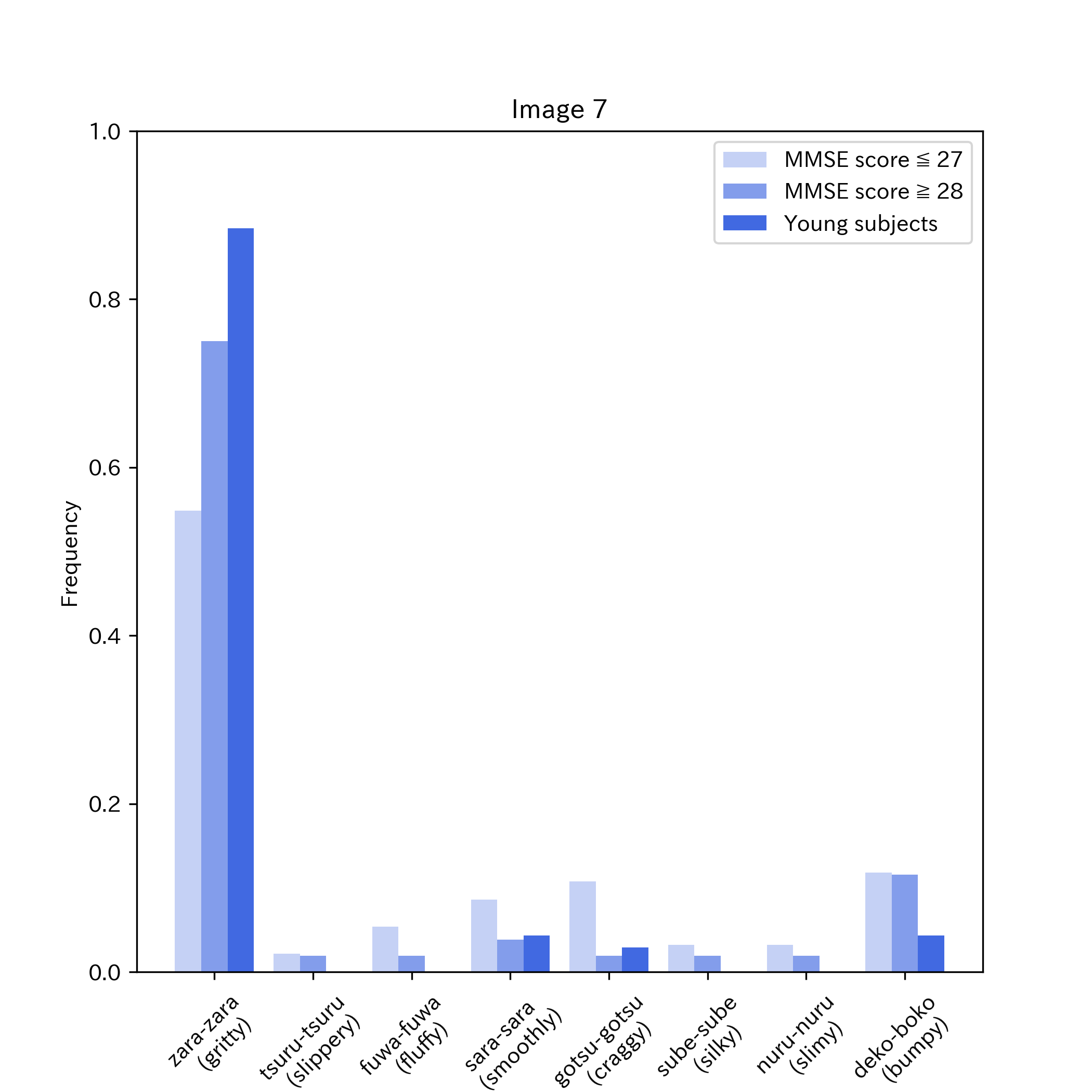

Supplement: Supplementary file 1 [file Supplementary_file_1.zip › S/FigS3g.jpg]

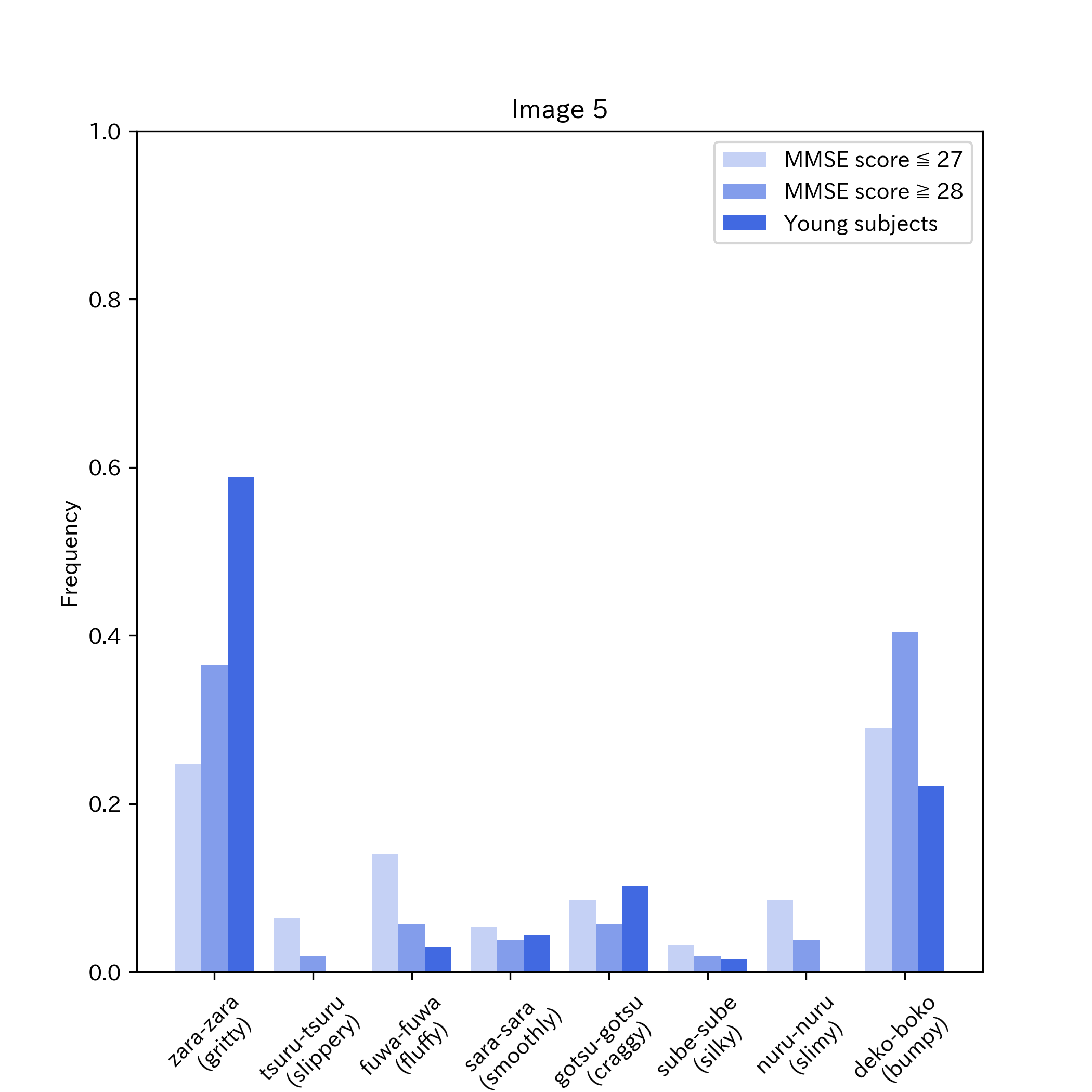

Supplement: Supplementary file 1 [file Supplementary_file_1.zip › S/FigS3e.jpg]

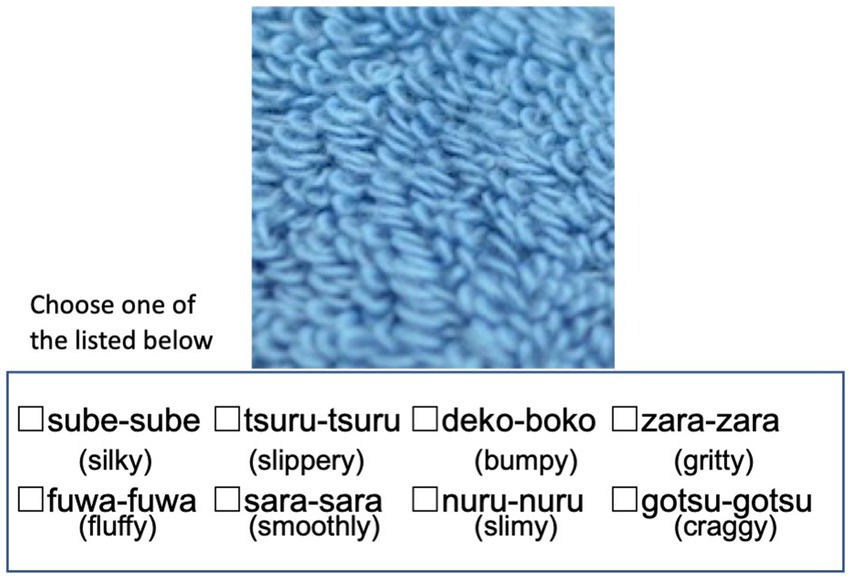

Supplement: Supplementary file 1 [file Supplementary_file_1.zip › S/FigS2.jpg]

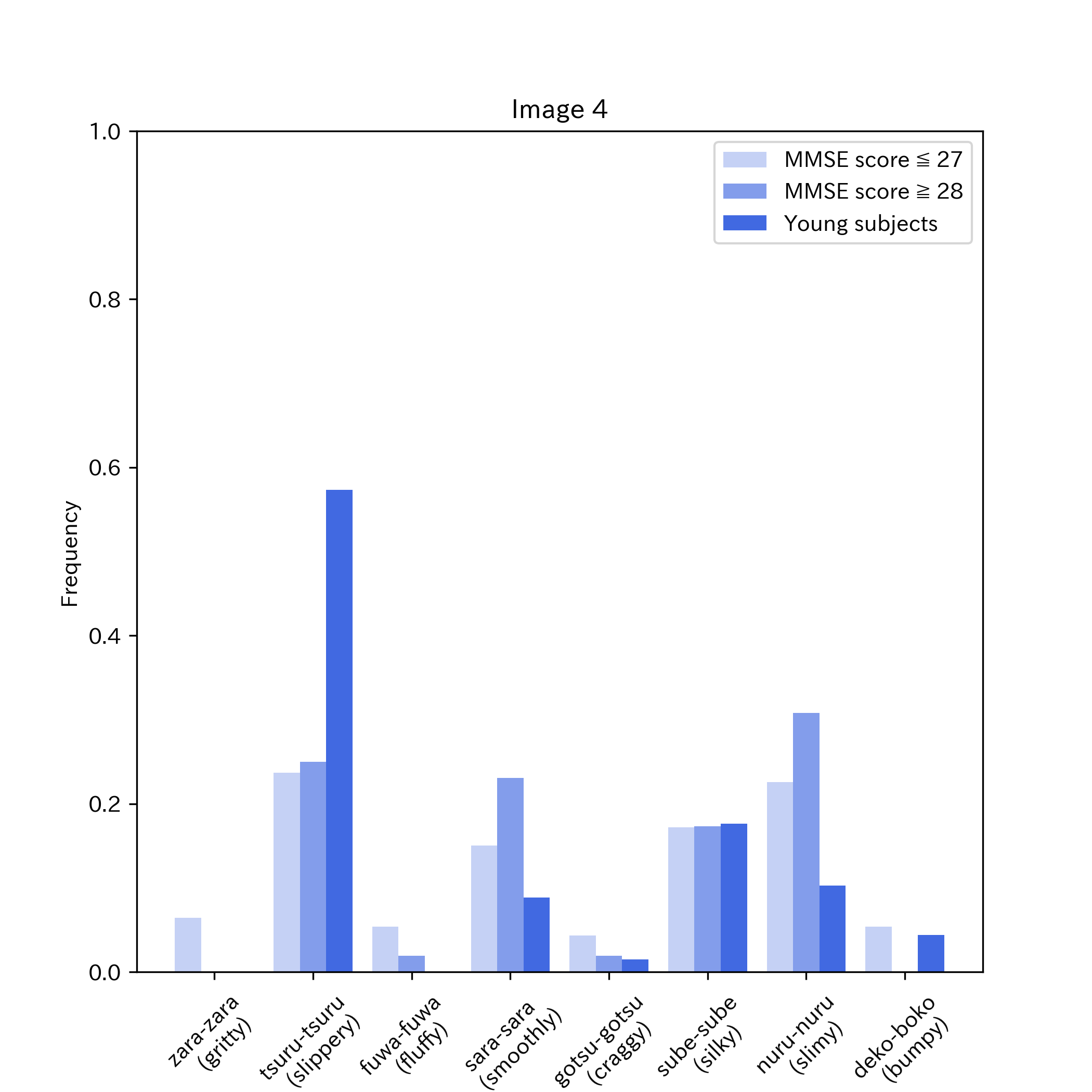

Supplement: Supplementary file 1 [file Supplementary_file_1.zip › S/FigS3d.jpg]

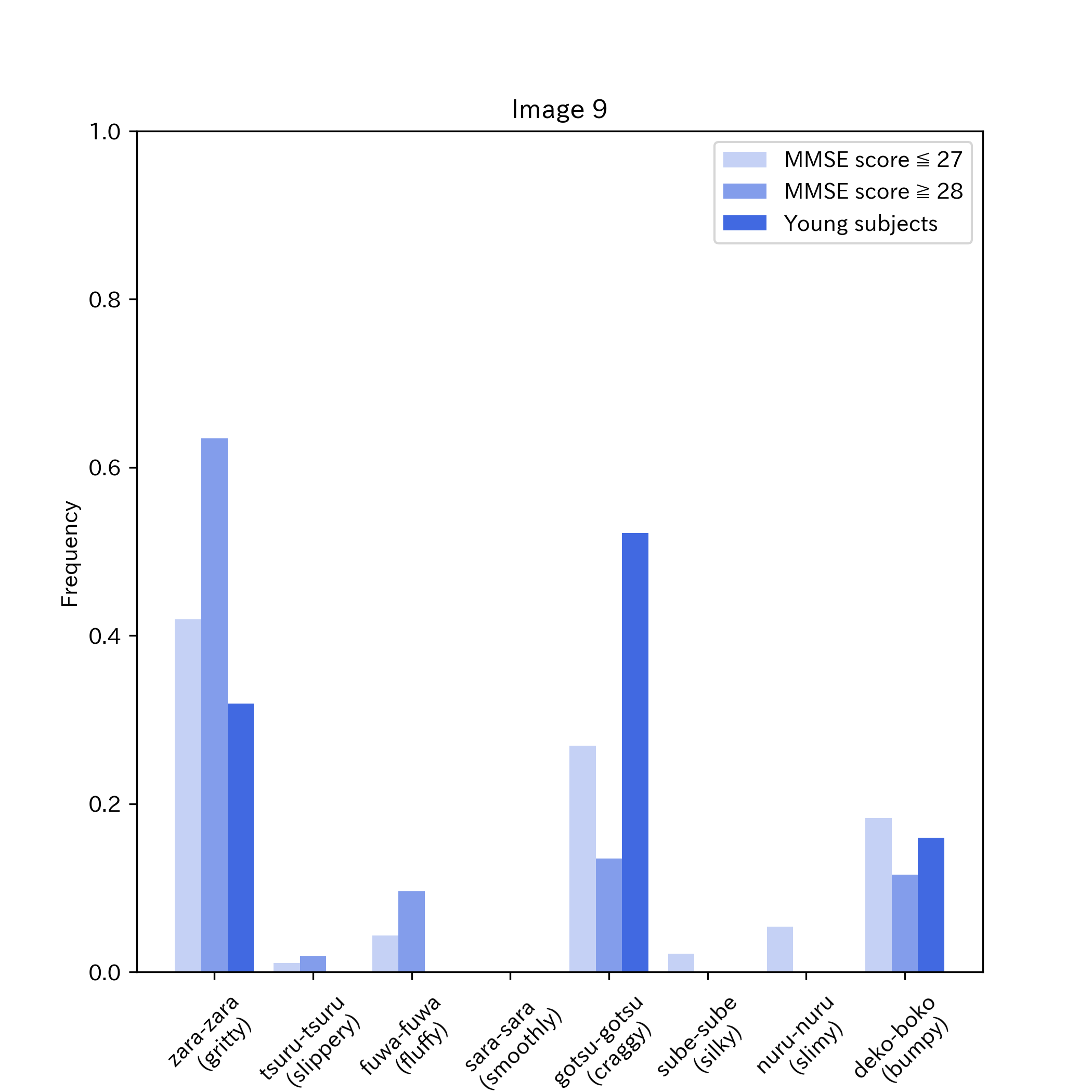

Supplement: Supplementary file 1 [file Supplementary_file_1.zip › S/FigS3i.jpg]

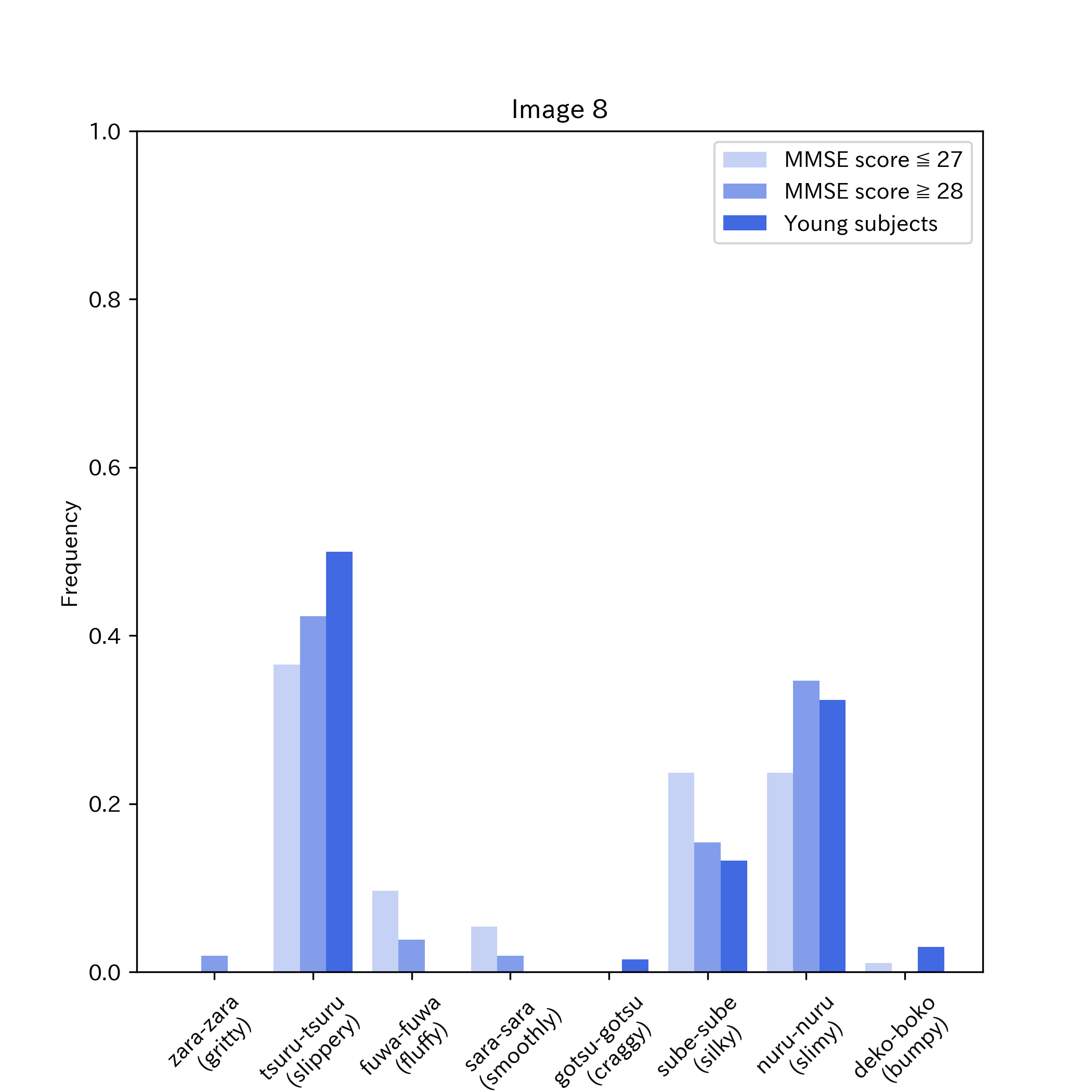

Supplement: Supplementary file 1 [file Supplementary_file_1.zip › S/FigS3h.jpg]

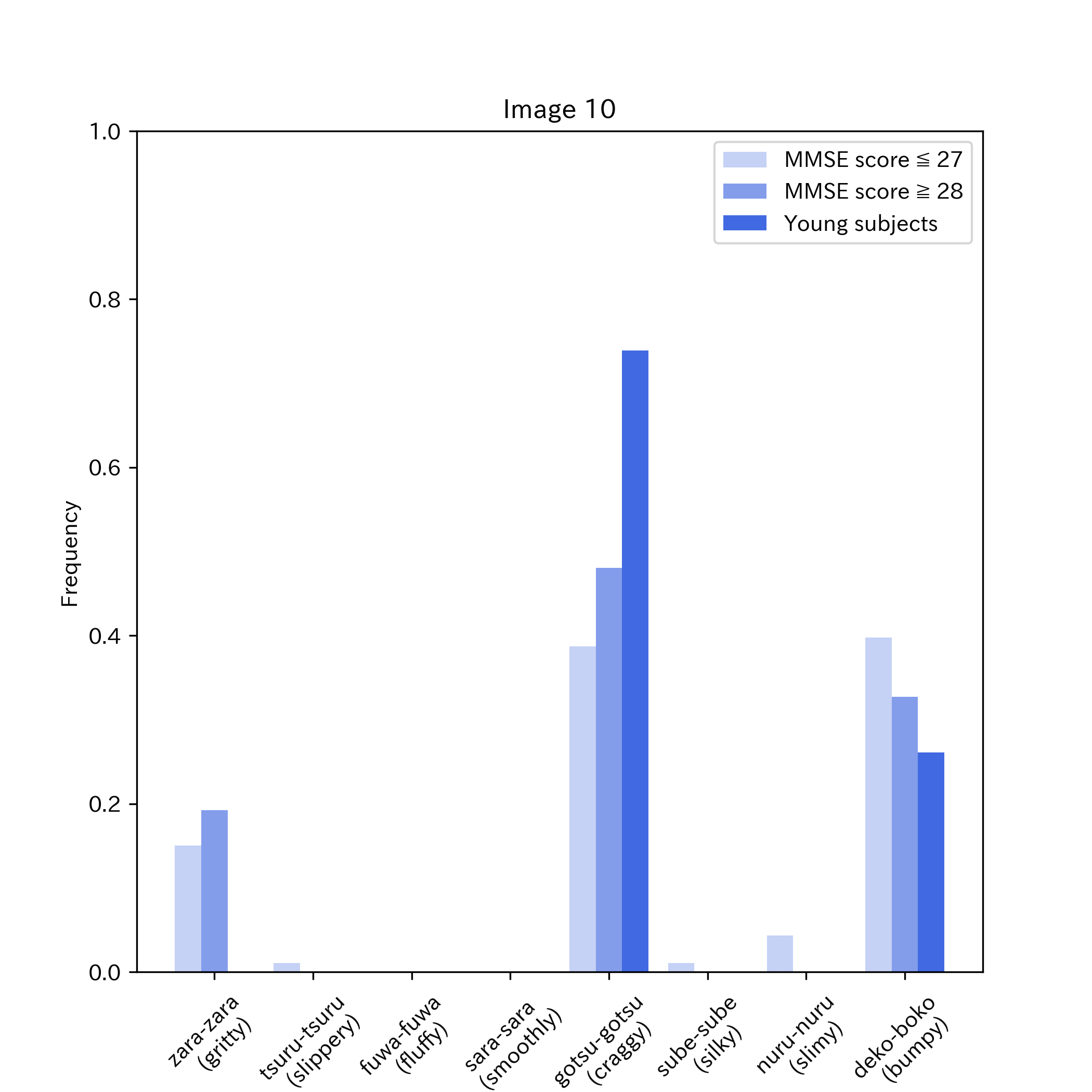

Supplement: Supplementary file 1 [file Supplementary_file_1.zip › S/FigS3j.jpg]

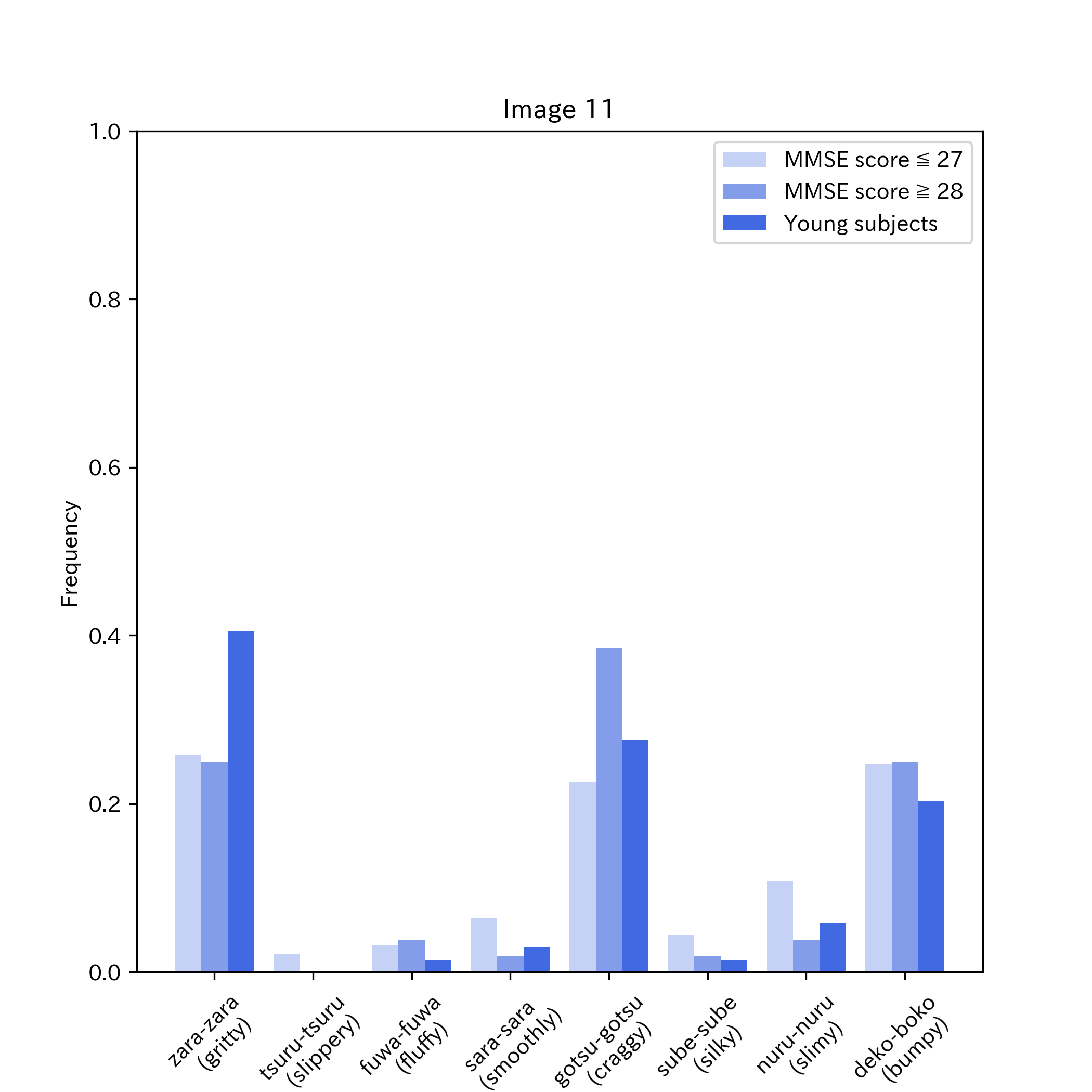

Supplement: Supplementary file 1 [file Supplementary_file_1.zip › S/FigS3k.jpg]

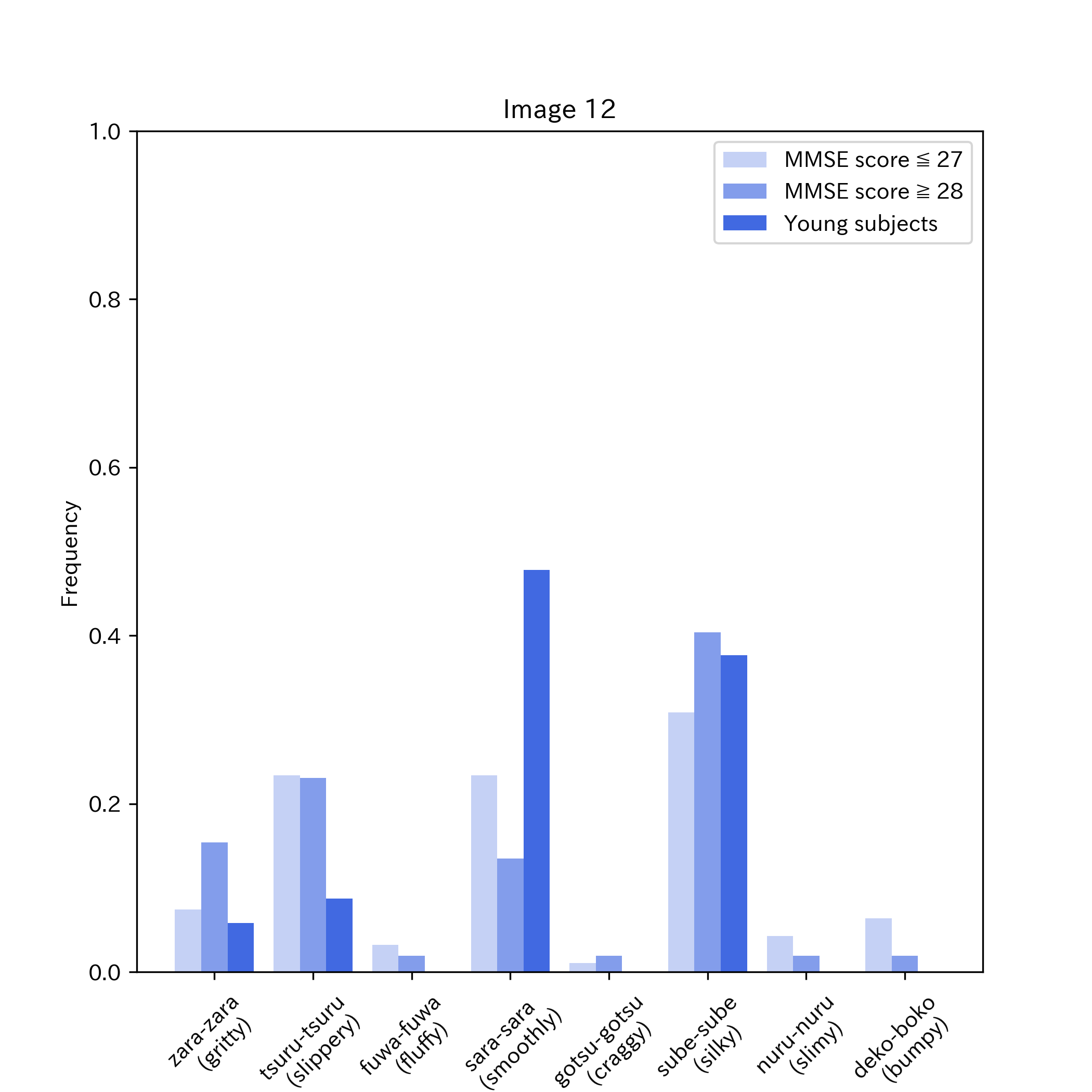

Supplement: Supplementary file 1 [file Supplementary_file_1.zip › S/FigS3l.jpg]
