# Supplementary material for: Machine learning-based detection of cognitive decline using SSWTRT: classification performance and decision analysis
Source: Front Artif Intell. 2025 Oct 29;8:1689182. doi: 10.3389/frai.2025.1689182 (PMC12605398; doi:10.3389/frai.2025.1689182)
Supplement: Supplementary file 2 [file Supplementary_file_2.docx]

Figure S1.
Twelve material surface images used in the SSWTRT, along with representative SSWs that express perceived texture. Adapted from Kamohara et al. (2024), Frontiers in Aging Neuroscience, 16:1456242, under CC BY 4.0 license.

Figure S2.
Procedure of the Sound Symbolic Words Texture Recognition Test (SSWTRT). Participants selected SSWs that best represented the texture of each presented material image. Adapted from Kamohara et al. (2024), Frontiers in Aging Neuroscience, 16:1456242, under CC BY 4.0 license.

Figure S3.
**Distributions of participants’ responses to the 12 image stimuli used in the test.** Panels (a)–(l) correspond to Images 1–12, respectively.
